# Phenanthroline-based microporous organic polymer as a platform for an immobilized palladium catalyst for organic transformations†

**DOI:** 10.1039/c9ra00460b

**Published:** 2019-03-13

**Authors:** Chang-An Wang, Kun Nie, Guo-Dong Song, Yan-Wei Li, Yin-Feng Han

**Affiliations:** College of Chemistry and Chemical Engineering, Taishan University Tai'an Shandong 271000 P. R. China wangcha@tsu.edu.cn han@tsu.edu.cn; Weifang University of Science and Technology, Shandong Peninsula Engineering Research Center of Comprehensive Brine Utilization Weifang 262700 P. R. China

## Abstract

Porous organic polymers have attracted significant attention owing to their large specific surface area, excellent chemical and thermal stability, and controllable skeletons. phenanthroline-based microporous organic polymer (Phen-MOP) has been synthesized *via* a cost-effective method based on the Scholl reaction. The Phen-MOP polymer exhibits high surface area and good stability. Owing to the phenanthroline skeleton embedding into the microporous polymer framework, the Phen-MOP can serve as a platform to support a transition metal catalyst. After being post-modified with palladium acetate, the synthesized Phen-Pd-MOP framework can serve as a highly efficient heterogeneous catalyst for the Suzuki–Miyaura coupling reaction and the Heck coupling reaction. Moreover, the Phen-Pd-MOP catalyst could be reused at least 10–12 times without any significant loss of the catalytic activity.

## Abstract 2

Phenanthroline-based microporous organic polymer (Phen-MOP) is synthesized *via* a cost-effective method based on the Scholl reaction. After post-modification with Pd(OAc)_2_, the synthesized Phen-Pd-MOP is a highly efficient heterogeneous catalyst for C–C coupling reactions.

## Graphical abstract

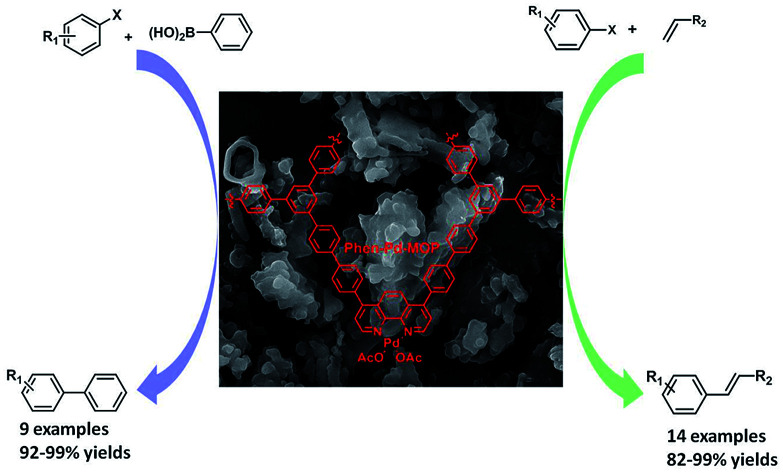

## Introduction

Porous organic polymers (POPs)^1^ with their high surface area and easily functionalization have diverse potential applications in the fields of gas storage/separation,^2^ light-harvesting,^3^ and heterogeneous catalysis.^4^ During the past decade, various POPs, such as conjugated microporous polymers (CMPs),^5^ hypercrosslinked polymers (HCPs),^6^ polymers of intrinsic microporosity (PIMs),^7^ covalent organic framework (COFs),^8^ and porous aromatic framework (PAFs),^9^ have been successfully designed and synthesized based purely on structural and functional organic building blocks. However, many expensive and rare transition/noble metal catalysts are used in constructing POPs materials, and functional groups (*e.g.* –Br, –I, –NH_2_, –C≡CH, –CHO, –CN, and –B(OH)_2_) are demanded in the organic building blocks for the synthesis of POPs frameworks, which hinders their practical application on a large scale. Accordingly, it is essential to develop a cost-effective method to synthesize functional POPs frameworks.

In this context, recently, Tan's group^10^ and Zhu's group^11^ independently reported a cost-effective approach to synthesize POPs frameworks based on the Scholl reaction. This synthetic method forms a new aryl–aryl bond by eliminating two aryl-bound hydrogen atoms in the presence of Lewis acid AlCl_3_ as a catalyst. Compared to the traditional polymerization reactions (expensive and rare transition/noble metal as the catalysts) used for the synthesis of CMPs, PAFs, or other POPs frameworks, the Scholl coupling reaction (AlCl_3_ as the catalyst) used for constructing POPs frameworks is economical and abundant, although the amount of catalyst required is equivalent. More importantly, there is no need for the design and synthesis of organic building blocks with special functional groups (*e.g.* –Br, –I, –NH_2_, –C≡CH, –CHO, –CN, and –B(OH)_2_) used in the synthesis of POPs frameworks. It means that we can save a lot of costs by avoiding tedious synthesis steps to prepare monomers. The microporous polymers formed by the Scholl reaction with the microporous and conjugated structure exhibit excellent properties for potential applications in the area of gas storage, separation, sensor, luminescence and semiconductor materials.

**Scheme 1:**
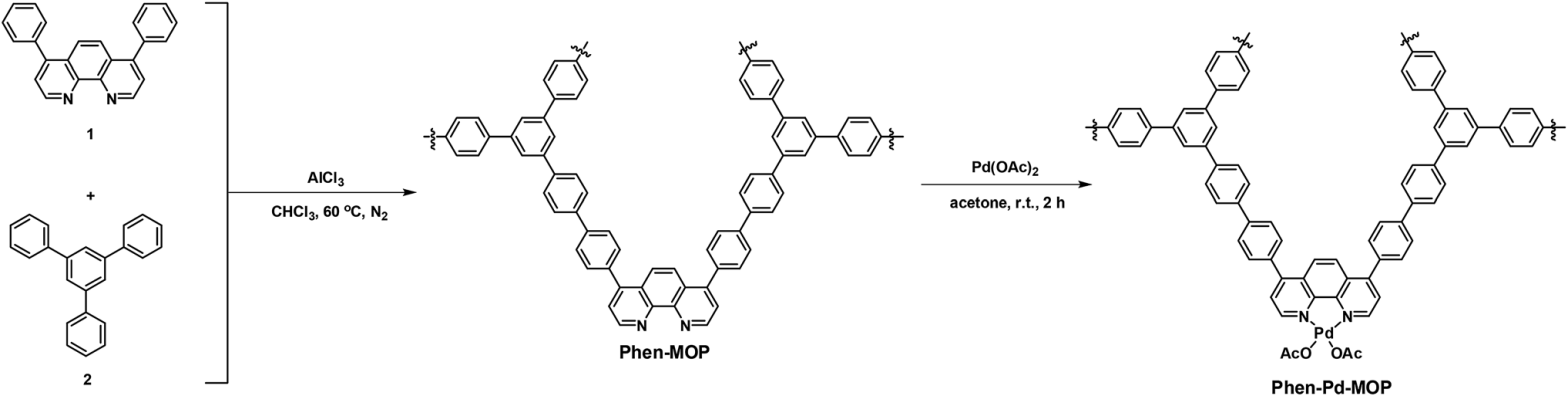
Synthesis of phenanthroline– and Pd(ii)–phenanthroline-based microporous organic polymers (Phen-MOP and Phen-Pd-MOP).

Phenanthroline, which is a nitrogen donor-based chelating ligand, has been extensively employed in coordination chemistry.^12^ In particular, owing to the robust redox stability, easy functionalization, and favored coordinate with metal ions,^13^ metal–phenanthroline ligands have been routinely used as homogeneous catalysts for organic transformations.^14^ For example, Yu's group reported a series of Pd-catalyzed C-3 selective C–H activations of pyridines with 1,10-phenanthroline as the ligand.^15^ However, these homogeneous catalysts are quite expensive and it is difficult to separate the catalysts from the reaction system, which limits their practical application. Therefore, exploration of heterogeneous catalysts based on phenanthroline and phenanthryl-derived ligands has attracted significant attention.^16^ In this context, Lin and co-workers reported the synthesis of a series of robust and porous phenanthryl-based metal–organic frameworks (MOFs) as highly active single site solid catalysts for tandem catalytic organic transformation.^17^ In consideration of the rigid skeleton structure and strong coordinating ability of phenanthroline, herein, we report a cost-effective strategy to synthesize phenanthroline-based microporous organic polymers based on the Scholl coupling reaction. The resulting POPs, denoted Phen-MOP, exhibit high surface areas and high thermal and chemical stability. After being post-modified with palladium ions, this porous polymer framework (denoted as Phen-Pd-MOP, Scheme 1) could be applied as a highly efficient and recyclable heterogeneous catalyst for organic transformations, such as the Suzuki–Miyaura coupling reaction and the Heck coupling reaction.^18^

## Results and discussion

### Synthesis and characterization of Phen-MOP and Phen-Pd-MOP

**Fig. 1:**
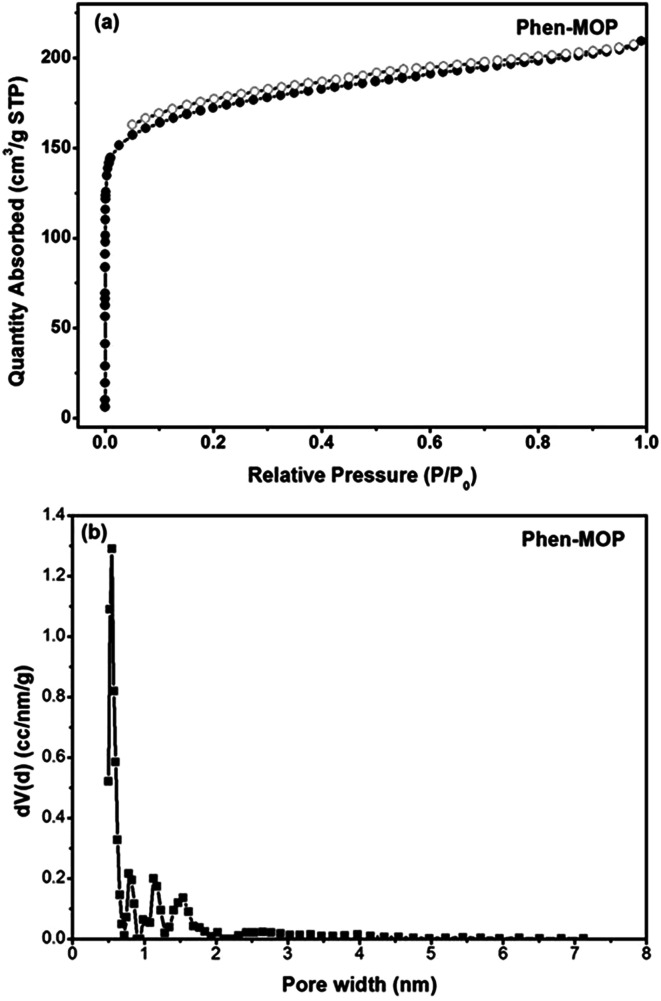
(a) N_2_ sorption isotherms measured at 77 K. (b) Pore size distribution (PSD) curve for Phen-MOP calculated by NLDFT method.

In order to realize the industrial application of functional POPs frameworks, the key factors for synthesizing POPs should be considered as follows: (i) the synthetic method should be easy to operate; (ii) the catalyst used must be low-cost; (iii) the polymers can be prepared on a large scale. Inspired by the pioneering work of Tan's group,^10^ in which multifunctional microporous organic polymers were easily synthesized based on the Scholl coupling reaction, we have selected 4,7-diphenyl-1,10-phenanthroline 1 as the functional building monomer and 1,3,5-triphenylbenzene 2 as the structural building monomer to synthesize a phenanthroline-based microporous organic polymer (Phen-MOP) *via* this cost-effective approach (Scheme 1). The obtained Phen-MOP framework was precipitated from the solution as a brown powder, and it was insoluble in all organic solvents tested owing to the highly cross-linked structure. The porosity of Phen-MOP was investigated by physisorption of nitrogen at 77 K. The representative curve of Phen-MOP exhibits a type I nitrogen gas sorption isotherm (Fig. 1(a)), which suggests that there are abundant micropores in the polymer. Based upon the calculations of the non-local density functional theory (NLDFT), the pore size distribution (PSD) of Phen-MOP is distributed around 0.5–1.5 nm (Fig. 1(b)). The Brunauer–Emmett–Teller (BET) surface area of Phen-MOP is estimated as 655 m^2^ g^−1^ with a total pore volume of 0.32 cm^3^ g^−1^.

**Fig. 2:**
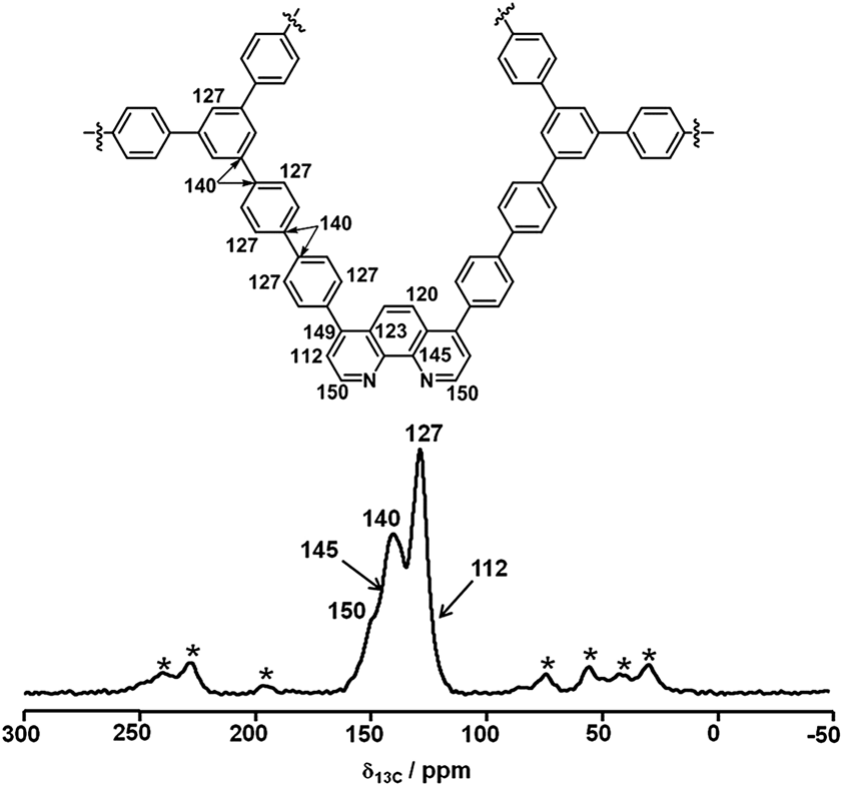
Cross-polarization (CP) ^13^C MAS NMR spectrum of Phen-MOP.

The structure of Phen-MOP was initially characterized by solid-state ^13^C cross-polarization magic-angle spinning (CP/MAS) NMR analysis (Fig. 2). The ^13^C CP/MAS NMR analysis confirmed the successful growth of a microporous network. As shown in Fig. 2, the resonance peaks at *δ* = 150, 145, 123, and 112 ppm can be assigned to the 1,10-phenanthroline skeleton, which suggests that the functional structure has been successfully embedded into the framework. The peaks at *δ* = 140 and 127 ppm can be assigned to the 1,3,5-triphenylbenzene skeleton. Additionally, elemental analysis of Phen-MOP identified the nitrogen content (2.34%), and the phenanthroline loading could be calculated based on the nitrogen content. All of the results confirmed the successful synthesis of the Phen-MOP framework.

**Fig. 3:**
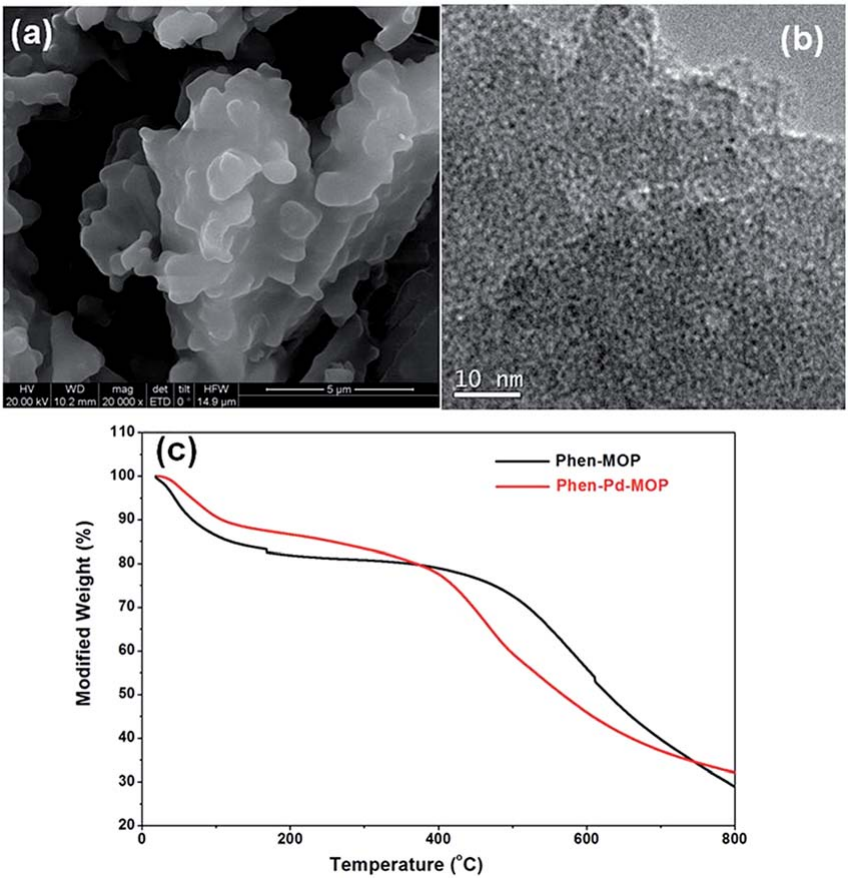
(a) SEM image of Phen-MOP. (b) TEM image of Phen-MOP. (c) TGA curves of Phen-MOP and Phen-Pd-MOP.

Scanning electron microscopy (SEM) revealed that the polymer consists of plate-shaped monoliths of several micrometers in size (Fig. 3(a)). High-resolution TEM image showed that abundant nanometer-scale cavities were present in the Phen-MOP framework (Fig. 3(b)). Thermogravimetric analysis (TGA) showed that the decomposition of the framework starts at 450 °C under a nitrogen atmosphere (Fig. 3(c)) and the modified weight (%) changed so much at the low temperature of 100 °C owing to these polymers adsorbing water when they are exposed to moist air for a long time. The PXRD patterns indicate that the Phen-MOP framework is amorphous in nature (Fig. S3†), the same as for previous POPs reported from our laboratory.^19^

**Fig. 4:**
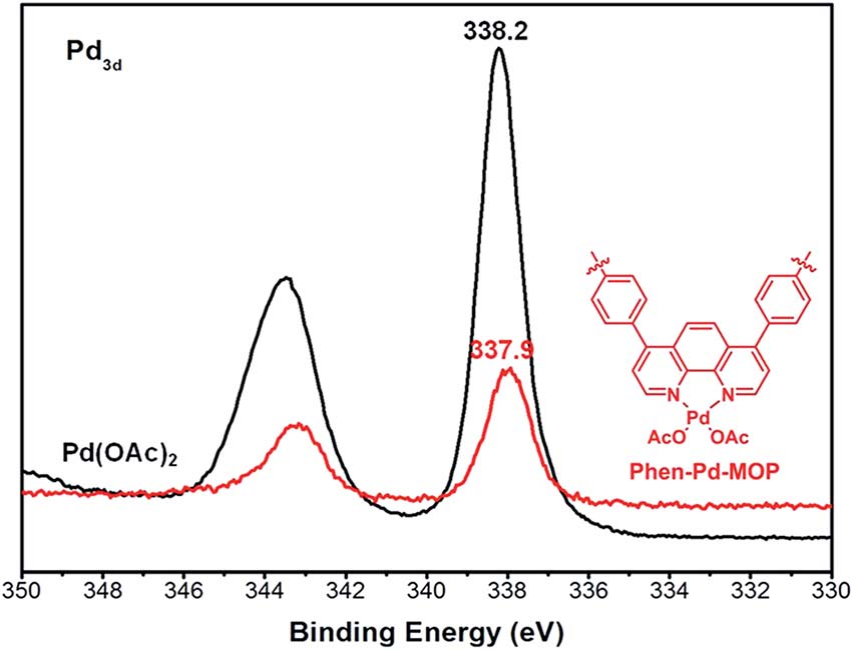
XPS spectra of Phen-Pd-MOP (red) and free Pd(OAc)_2_ (black).

**Fig. 5:**
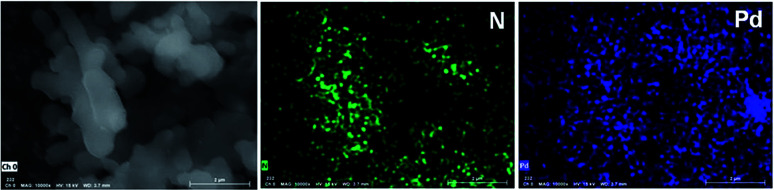
EDX elemental mapping of Phen-Pd-MOP.

The Phen-MOP framework, with bidentate nitrogen-binding sites, was simply post-treated with Pd(OAc)_2_ to yield the palladium-incorporated porous organic catalyst Phen-Pd-MOP. TGA showed that Phen-Pd-MOP was stable up to around 350 °C under a nitrogen atmosphere (Fig. 3(c)). Compared with the Phen-MOP framework, the BET surface area of Phen-Pd-MOP decreased to 403 m^2^ g^−1^ (Fig. S1 in the ESI†), the reason is that the Pd(OAc)_2_ is handing in the nanopores volume. Next, we selected X-ray photoelectron spectroscopy (XPS) measurements and energy-dispersive X-ray spectroscopy (EDX) to survey the coordination of palladium within the Phen-Pd-MOP framework. As shown in Fig. 4 (red), the binding energy (BE) of Pd_3d5/2_ in Phen-Pd-MOP is 337.9 eV, which indicated that the Pd species in the Phen-Pd-MOP framework was present in a 2+ oxidation state. In comparison with the BE of 338.2 eV for free Pd(OAc)_2_ (Fig. 4 (black)), the Pd(ii) BE in Phen-Pd-MOP was shifted negatively by 0.3 eV, which indicated the strong coordination of Pd(OAc)_2_ with the bidentate nitrogen group of Phen-MOP. We also found that the BE of Pd_3d5/2_ in Phen-Pd-MOP has the same value in Pd/Phen (ref. 20) (this structure was synthesized from 1,10-phenanthroline and Pd(OAc)_2_). The chemical state of N elements in Phen-MOP and Phen-Pd-MOP has also been investigated by XPS analysis (Fig. S2 in the ESI†). As shown in Fig. 5, elemental mapping using energy-dispersive X-ray spectroscopy (EDX) showed that a homogeneous distribution of palladium accompanied by the well-dispersed element nitrogen were found in the Phen-Pd-MOP framework. All of the results confirm that the Pd(ii) is successfully immobilized on the Phen-MOP by coordination to phenanthroline functional groups rather than by physical adsorption of Pd(OAc)_2_ on the surface.

### Heterogeneous catalytic application of Phen-Pd-MOP

After the palladium-incorporated porous polymers were synthesized and characterized, we then investigated the catalytic activity of the Phen-Pd-MOP framework as a robust and powerful heterogeneous catalyst. The resultant Phen-Pd-MOP was firstly tested on the Suzuki–Miyaura coupling reaction of arylboronic acids with various types of aryl halides in different solvents. We chose the reaction of phenylboronic acid with bromobenzene as the model reaction to optimize the reaction conditions, and the results are listed in Table S1 (see the ESI†). As shown in Table S1,† the reaction did not work when the Phen-MOP framework was used as the catalyst or in the absence of Phen-Pd-MOP, which confirmed that the Phen-Pd-MOP played the catalytic role (Table S1, entries 10 and 11†). Screening experiments with different solvents revealed that the Suzuki–Miyaura reaction gave the highest yield when performed in EtOH/H_2_O (1 : 1, v/v) at 80 °C (Table S1, entry 8†). Under the optimized conditions, we then examined the scope of the Phen-Pd-MOP-catalyzed Suzuki–Miyaura reaction between phenylboronic acid and various bromo- and iodo-benzene derivatives. As shown in Table 1, all of the reactions were completed very efficiently, giving excellent yields of the corresponding products. For the aryl bromide derivatives, regardless of the effects of electron-donating or -withdrawing functions, high catalytic activity was observed (Table 1, entries 1–7). Especially for aryl iodides, the coupling reactions were rapidly completed within 0.5 h and gave high yields (entries 8 and 9). Next, in order to determine that the reaction was indeed catalyzed by the Phen-Pd-MOP catalyst and not the dissolved homogeneous Pd species leached from the supports, the following strategy was adopted. When the phenylboronic acid conversion reached about 50%, the reaction mixture was quickly centrifuged to remove the Phen-Pd-MOP catalyst and then the hot mother liquor was allowed to react for another 2 h under similar conditions. No significant changes were observed in either the conversion or the yield, indicating that the catalytic species was not dissolved Pd(ii) leached from the Phen-Pd-MOP framework.

**Table 1:** Phen-Pd-MOP-catalyzed Suzuki–Miyaura reaction of various aryl halides with phenylboronic acid^a^

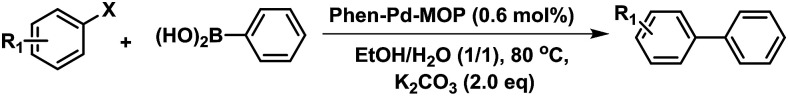				
Entry	Ar–X	Time (h)	Yield^b^ (%)	TON^c^
1^d^	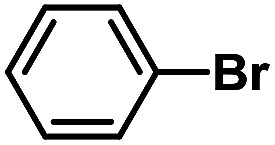	0.5	99	165
2	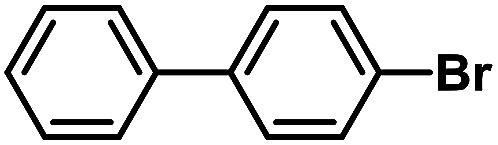	8	95	158
3	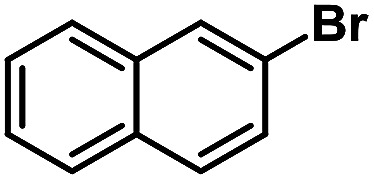	2	97	162
4	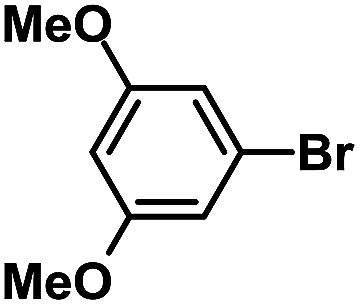	6	94	157
5	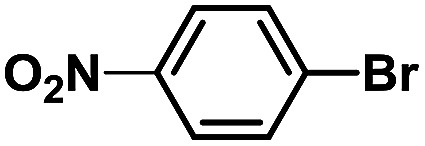	5	93	155
6	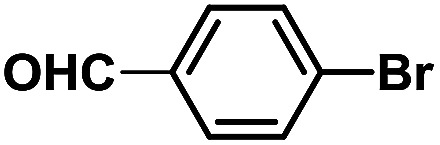	2	92	153
7	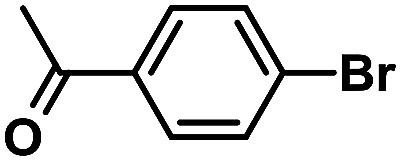	2	97	162
8^d^	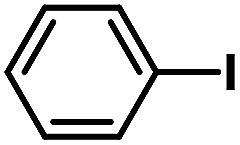	0.5	99	165
9	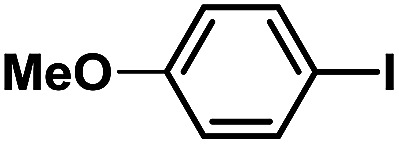	0.5	98	196

a Reaction conditions: aryl halide (0.5 mmol), phenylboronic acid (0.75 mmol), K_2_CO_3_ (1.0 mmol), Phen-Pd-MOP (0.6 mol%), EtOH/H_2_O (1.0 mL), 80 °C.

b Isolated yield after silica gel column chromatography.

c TON = (moles of product)/(moles of Pd in the catalyst).

d Aryl halide (0.75 mmol), phenylboronic acid (0.5 mmol).

In order to verify the universality of the Phen-Pd-MOP catalyst, we then chose the palladium-catalyzed Heck coupling reaction to further investigate the catalytic activity of the Phen-Pd-MOP framework. Firstly, we selected the Heck coupling reaction of iodobenzene with methyl acrylate to optimize the reaction conditions, and the results are listed in Table S3 (see the ESI†). Screening experiments with different solvents and temperatures showed that the best reaction conditions were: DMF as the solvent, 0.6 mol% Phen-Pd-MOP as the catalyst, in the presence of Et_3_N, and at 130 °C (Table S3, entry 1†). With the best reaction conditions established, various aryl iodides and bromides were tested to examine the scope of the Heck coupling reaction, and the results are shown in Table 2. For both electron-donating aryl iodides (entries 1–4) and electron-withdrawing aryl iodides (entries 5 and 6), as well as sterically hindered aryl iodides (entries 7–9), the reaction proceeded efficiently to give the corresponding products in excellent yields (94–99%) within 2 h. However, for aryl bromides (entries 10–12), the reaction proceeded more slowly to afford the products in good to excellent yields (89–94%). With styrene as a substrate, the reaction also afforded the corresponding products with high yields, although a longer reaction time was required (entries 13 and 14). These results showed that the Phen-Pd-MOP framework could work as a highly efficient heterogeneous catalyst for the Suzuki–Miyaura reaction and the Heck coupling reaction.

**Table 2:** Phen-Pd-MOP catalyzed the Heck coupling reaction of various aryl halides to olefin^a^

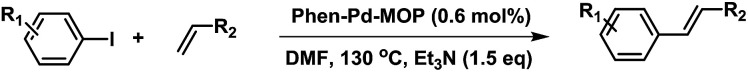					
Entry	Aryl halide	Olefin	Time (h)	Yield^b^ (%)	TON^c^
1	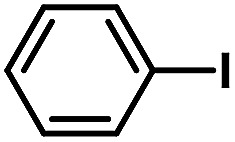	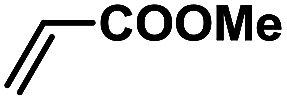	1	99	165
2	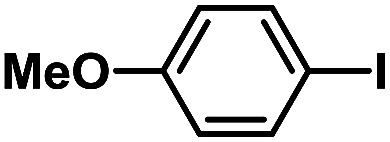	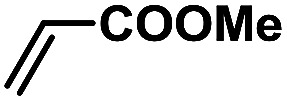	2	95	158
3	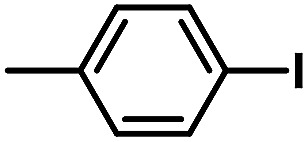	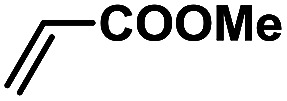	2	96	160
4	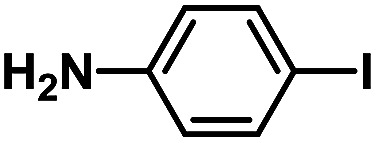	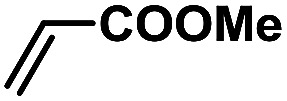	2	99	165
5	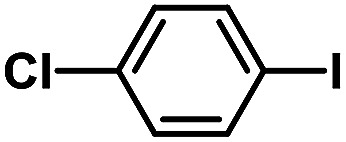	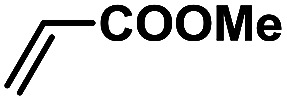	2	99	165
6	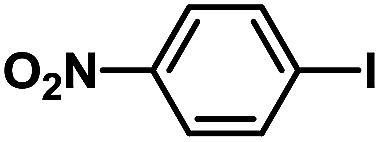	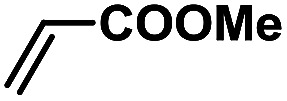	2	97	162
7	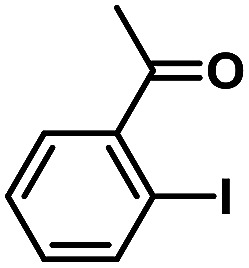	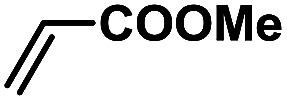	2	95	158
8	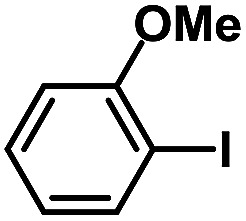	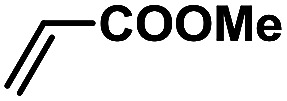	1	98	163
9	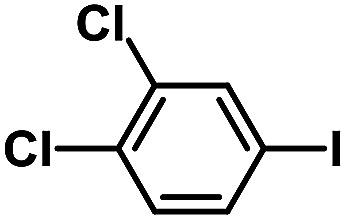	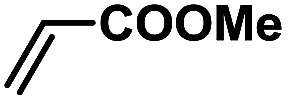	1	94	157
10	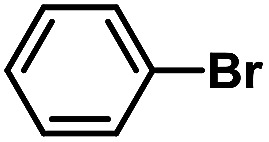	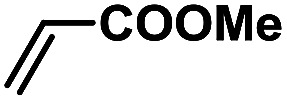	5	92	153
11	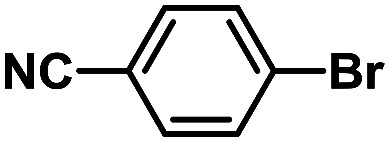	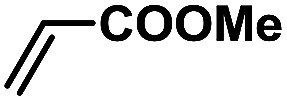	5	89	148
12	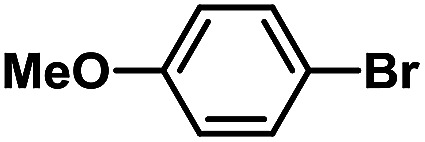	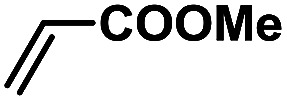	5	94	157
13	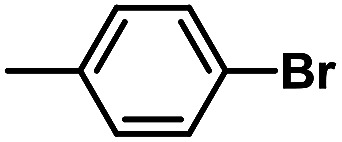	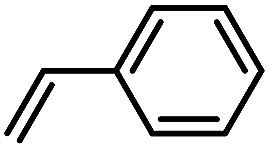	24	80	133
14	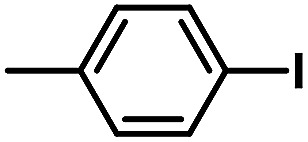	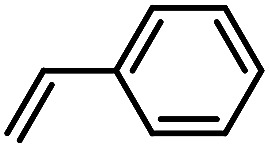	15	82	137

a General conditions: aryl halide (0.5 mmol), olefin (0.75 mmol), Et_3_N (0.75 mmol, 1.5 eq.), DMF (1.0 mL) and Phen-Pd-MOP (0.6 mol%), 130 °C.

b Isolated yields.

c TON = (moles of product)/(moles of Pd in the catalyst).

**Fig. 6:**
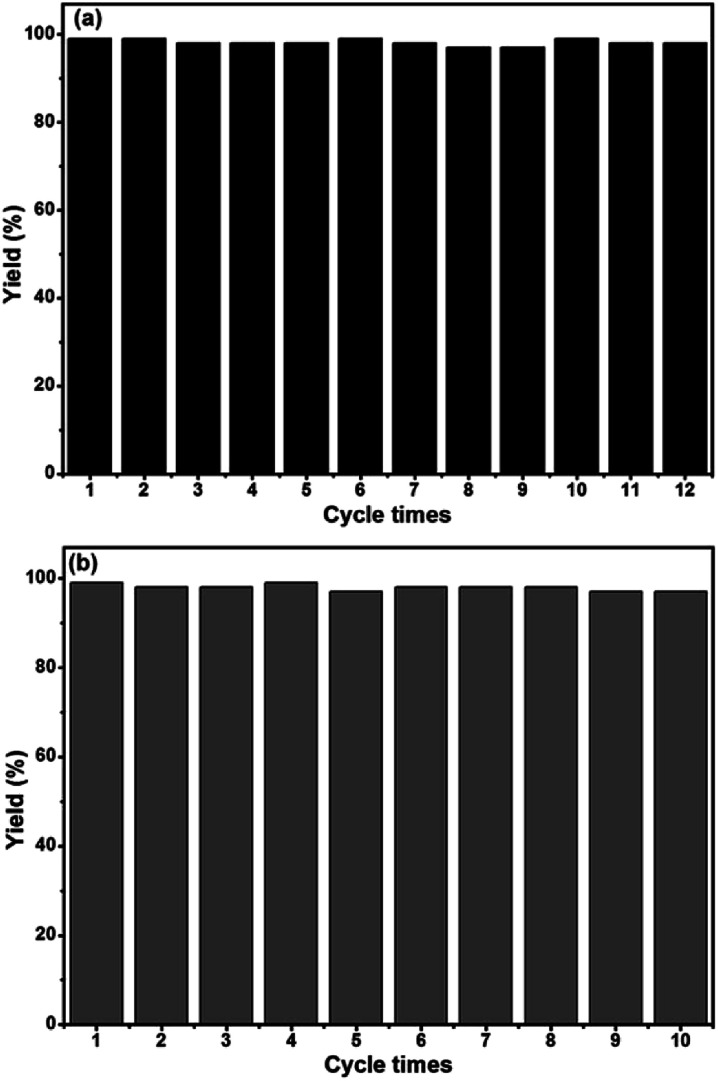
Phen-Pd-MOP recycling experiments for the Suzuki–Miyaura coupling reaction (a) and Heck coupling reaction (b).

The recyclability is a significant indicator for evaluating robust and powerful heterogeneous catalysts. Therefore, the recyclability of the Phen-Pd-MOP catalyst was also investigated. The catalytic recyclability of Phen-Pd-MOP was estimated by examining both the Suzuki–Miyaura reaction of phenylboronic acid with bromobenzene (see ESI, Table S2†) and the Heck coupling reaction of iodobenzene with methyl acrylate (see ESI, Table S4†). The recycling experiment was performed by recovering the Phen-Pd-MOP by using a simple centrifugation method and the recovered catalyst was then washed with EtOH and EtOAc to remove the adherent products. After being dried under vacuum in 80 °C, the heterogeneous catalyst could be reused directly without further purification. As shown in Fig. 6, owing to the strong coordination ability of Phen-MOP and the highly dispersed Pd(ii) ions embedded into the Phen-MOP framework, this Phen-Pd-MOP catalyst could be recycled and reused at least 12 times for the Suzuki–Miyaura coupling reaction or 10 times for the Heck coupling reaction without loss of the catalytic activity. Meanwhile, we did not find any Pd(ii) ions leaching from the Phen-Pd-MOP catalyst after every cycle, as determined by ICP. The BET surface area of the Phen-Pd-MOP framework decreased to 272 m^2^ g^−1^ after the 12^th^ cycle of use (Fig. S1 in the ESI†), which could be due to the partial blocking of the polymeric nanopores by substrates.

## Conclusions

In summary, we have reported a new and cost-effective strategy to synthesize a phenanthroline-functionalized microporous organic polymer (Phen-MOP) based on the Scholl coupling reaction. The new polymer has outstanding stability and good porosity with a high BET surface area. Owing to the phenanthroline skeleton embedded in the microporous polymer framework, Phen-MOP can serve as an ideal platform to support transition metal catalysts for heterogeneous catalysis. After being post-modified with palladium acetate, the synthesized Phen-Pd-MOP framework can serve as a highly efficient heterogeneous catalyst for a wide range of organic transformations. Catalyzed by the Phen-Pd-MOP catalyst, the Suzuki–Miyaura coupling reaction of aryl halides to phenylboronic acid gave the corresponding products in excellent yields (92–99%) and it could be reused at least 12 times without loss of catalytic activity. Meanwhile, catalyzed by the Phen-Pd-MOP catalyst, the Heck coupling reaction of aryl halides to olefin also gave the desired products in good to excellent yields (82–99%) and it could be recycled and reused 10 times without an obvious decrease in the catalytic efficiency. We believe that the low-cost Phen-MOP framework prepared in this work may act as an ideal platform to support transition metal catalysts for application on a large scale.

## Experimental section

### Materials

4,7-Diphenyl-1,10-phenanthroline and 1,3,5-triphenylbenzene were received from J&K. Pd(OAc)_2_ and chloroform and anhydrous AlCl_3_ were purchased from Energy Chemical (Shanghai, China) and used as received. All reagents were obtained from commercial suppliers and used without further purification. All catalytic reactions were performed in a 15 mL pressure tube.

### Synthesis of Phen-MOP

Anhydrous AlCl_3_ (665 mg, 5.0 mmol) was added to a 100 mL round-bottomed flask. Then, after pumping to vacuum, the system was inflated with inert gas N_2_ three times. Next, 20 mL of dried CHCl_3_ was injected through a syringe and the mixture was heated to 60 °C for 30 min. Then 4,7-diphenyl-1,10-phenanthroline (166 mg, 0.5 mmol) and 1,3,5-triphenylbenzene (102 mg, 0.33 mmol) dissolved in 20 mL of dried CHCl_3_ were added into the system and the mixture was kept stirring at 60 °C for 24 h. After the reaction had completed, the crude product was obtained by filtration and washed with 1 M hydrochloric acid solution, methanol, and acetone to remove unreacted monomers and catalyst residues. Further purification of the polymer was carried out by Soxhlet extraction with methanol for 48 h. The polymer was dried at 80 °C under vacuum for 6 h to give a brown powder. Yield: 262 mg (98%). Elemental analysis (%) found: C 48.73, N 2.34, H 4.09.

### Synthesis of Phen-Pd-MOP catalyst

Palladium acetate (45 mg, 0.2 mmol) was dissolved in 10 mL of acetone, and then Phen-MOP (100 mg, 0.08 mmol) was added. The mixture was kept stirring for 2 h at room temperature. After the reaction was complete, the resulting solid was filtered and washed thoroughly with acetone (10 mL × 5), distilled water (10 mL × 5) and MeOH (10 mL × 5), then dried at 80 °C under vacuum for 12 h to yield Phen-Pd-MOP as a brown powder (110 mg). The Pd content in Phen-Pd-MOP was 10.60%, as determined by ICP.

### General procedure for the Suzuki–Miyaura reaction catalyzed by Phen-Pd-MOP

In a typical run of a catalytic activity test of Phen-Pd-MOP, aryl halides (0.5 mmol), phenylboronic acid (91.5 mg, 0.75 mmol, 1.5 eq.), K_2_CO_3_ (138 mg, 1.0 mmol, 2.0 eq.), and Phen-Pd-MOP (3.0 mg, 0.6 mol%) were added to 1.0 mL of 1.0/1.0 EtOH–H_2_O (v/v) mixture. The reaction mixture was stirred at 80 °C under an ambient atmosphere. After the reaction was completed (monitored by TLC), the mixture was centrifuged and the solid was washed with EtOH (1 × 5 mL) and EtOAc (3 × 5 mL). The combined organic phase was washed with water to remove K_2_CO_3_ residue. After the evaporation of the solvent under vacuum, the residue was purified by flash column chromatography with petroleum ether or petroleum ether–EtOAc = 10 : 1 as the eluent.

### General procedure for Heck coupling reaction catalyzed by Phen-Pd-MOP

In a typical run of a catalytic activity test of Phen-Pd-MOP for the Heck coupling reaction, aryl halides (0.5 mmol), methyl acrylate (64.5 mg, 0.75 mmol, 1.5 eq.), Et_3_N (0.75 mmol, 1.5 eq.), and Phen-Pd-MOP (3.0 mg, 0.6 mol%) were added to 1.0 mL of DMF. The reaction mixture was stirred at 130 °C under a N_2_ atmosphere. After the reaction was completed (monitored by TLC), the mixture was cooled to room temperature and centrifuged, and the obtained Phen-Pd-MOP catalyst was washed with EtOAc (3 × 5 mL). The combined organic phase was washed with water and dried over anhydrous Na_2_SO_4_. After the evaporation of the solvent under vacuum, the residue was purified by flash column chromatography with petroleum ether–EtOAc (6 : 1 to 20 : 1) as the eluent.

### Recyclability of Phen-Pd-MOP catalyst

The Phen-Pd-MOP catalyst was recycled by centrifugation method. The recovered Phen-Pd-MOP catalyst was washed with EtOH and EtOAc to remove the residual product and then simply dried before reuse. We chose the Suzuki–Miyaura coupling reaction of bromobenzene with phenylboronic acid and the Heck coupling reaction of iodobenzene with methyl acrylate to investigate the recyclability of the Phen-Pd-MOP catalyst, and the results are summarized in Tables S2 and S4.†

## Conflicts of interest

There are no conflicts to declare.

## Supplementary Material

RA-009-C9RA00460B-s001
