# Supplementary material for: Phenanthroline-based microporous organic polymer as a platform for an immobilized palladium catalyst for organic transformations
Source: RSC Adv. 2019 Mar 13;9(15):8239–45. doi: 10.1039/c9ra00460b (PMC9061877; doi:10.1039/c9ra00460b)
Supplement: RA-009-C9RA00460B-s001 [file RA-009-C9RA00460B-s001.pdf]

Electronic Supplementary Material (ESI) for RSC Advances.
This journal is © The Royal Society of Chemistry 2019

[Supporting Information]

Phenanthroline-Based Microporous Organic Polymer as a Platform Immobilized Palladium Catalyst for Organic Transformations

Chang-An Wang[a]*, Kun Nie[a], Guo-Dong Song[b], Yan-Wei Li[a], Yin-Feng Han [a]*

[a] *College of Chemistry and Chemical Engineering, Taishan University, Tai'an, Shandong 271000, P. R. China.*

[b] *Weifang University of Science and Technology, Shandong Peninsula Engineering Research Center of Comprehensive Brine Utilization, Weifang 262700, P. R. China.*

E-mail: wangcha@tsu.edu.cn; han@tsu.edu.cn

A. General information
B. Materials and experimental procedures
C. N_2 adsorption-desorption isotherms of **Phen-Pd-MOP** and **the** recycled **Phen-Pd-MOP**
D. N 1s XPS spectra for **Phen-MOP** and **Phen-Pd-MOP**
E. Powder X-Ray diffraction for **Phen-MOP**
F. Screening conditions for the Suzuki-Miyaura coupling reaction
G. General procedure for the Suzuki-Miyaura coupling reaction
H. Recyclability of **Phen-Pd-MOP** catalyst for the Suzuki-Miyaura coupling reaction
I. Screening conditions for Heck coupling reaction
J. General procedure for Heck coupling reaction
K. Recyclability of **Phen-Pd-MOP** catalyst for the Heck coupling reaction
L. ^{1}H NMR and ^{13}C NMR spectra of the products

A. General information

Chemicals and solvents were purchased from commercial suppliers. All equipment was thoroughly oven-dried. Thin-layer chromatography (TLC) plates were visualized by exposure to ultraviolet light. Flash column chromatography (FCC) was carried out with silica gel (200–300 mesh). ^{1}H and ^{13}C liquid NMR spectra were recorded on a Bruker Avance III 400 MHz NMR spectrometer. The chemical shifts δ are given in ppm (parts per million) relative to tetramethylsilane (TMS) and the coupling constants J are given in Hz. All the spectra were recorded in $CDCl_3$ as solvent at room temperature. TMS served as the internal standard (δ = 0.00 ppm) for ^{1}H NMR, while $CDCl_3$ as the internal standard (δ = 77.0 ppm) for ^{13}C NMR. Elemental analysis was performed on an Elementar Analysensysteme GmbH VarioEL V3.00 elemental analyzer. N_2 adsorption and desorption isotherms were measured at 77 K using a Quantachrome autosorb IQ-2. The pore-size-distribution curves were obtained from the adsorption branches using non-local density functional theory (NLDPT) method. Solid-state NMR experiments were performed on a Bruker Avance II WB 400 MHz NMR spectrometer. The ^{13}C CP/MAS NMR spectra were recorded with the contact time of 3 ms (ramp 100) and the recycle delay of 2 s on a 2.5 mm double resonance probe. Thermal properties of the synthesized materials were evaluated on a STA PT1600 Linseis thermogravimetric analysis (TGA) instrument in the temperature range of 25 to 800 °C under nitrogen atmosphere with a heating rate of 10 °C/min. The Pd contents in polymer frameworks were determined by Agilent ICP-OES. Surface morphologies and microstructures of the synthesized materials were examined with a Hitachi S-4800 scanning electron microscope (SEM) and with a JEOL JEM-2010 transmission electron microscope (TEM) operated at 200 kV. X-ray photoelectron spectroscopy (XPS) date were obtained with an Thermo ESCALAB 250XI Scientific electron spectrometer using 150W Al Kα radiation.

B. Materials and experimental procedures

Materials: 4,7-diphenyl-1,10-phenanthroline and 1, 3, 5-triphenylbenzene were received from J&K. $Pd(OAc)_2$ and Chloroform and anhydrous $AlCl_3$ were purchased from Energy Chemical (Shanghai, China) and used as received. All reagents were obtained from commercial suppliers and used without further purification. All catalytic reactions were performed in a 15 mL pressure tube.

Experimental procedures:

1) Synthesis of **Phen-MOP** materials[1]

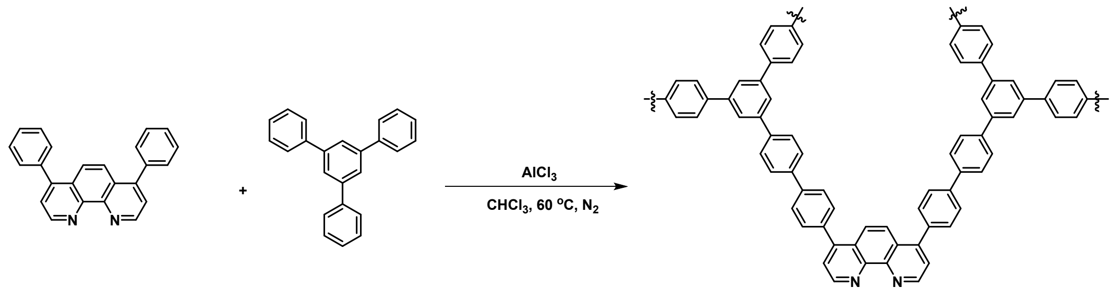

Anhydrous $AlCl_3$ (665 mg, 5.0 mmol) was added to a 100 mL round-bottomed flask. Then, after pumped to vacuum, the system was inflated with inert gas N_2 for 3 times. Next, 20 mL dried $CHCl_3$ was injected through a syringe and the mixture was heated to 60 °C for 30 min. Then 4,7-diphenyl-1,10-phenanthroline (166 mg, 0.5 mmol) and 1, 3, 5-triphenylbenzene (102 mg, 0.33 mmol) dissolved in 20 mL dried $CHCl_3$ were added into the system and the mixture kept stirring at 60 °C for 24 h. After the reaction completed, the crude product was obtained by filtration and washed with 1 M hydrochloric acid solution, methanol, and acetone to remove unreacted monomers and catalyst residues. Further purification of the polymer was carried out by Soxhlet extraction with methanol for 48 h. The polymer was dried at 80 °C under vacuum for 6 h to give a brown powder. Yield: 262 mg (98%). Elemental analysis (%) found: C 48.73, N 2.34, H 4.09.

2) Synthesis of **Phen-Pd-MOP** catalyst[2]

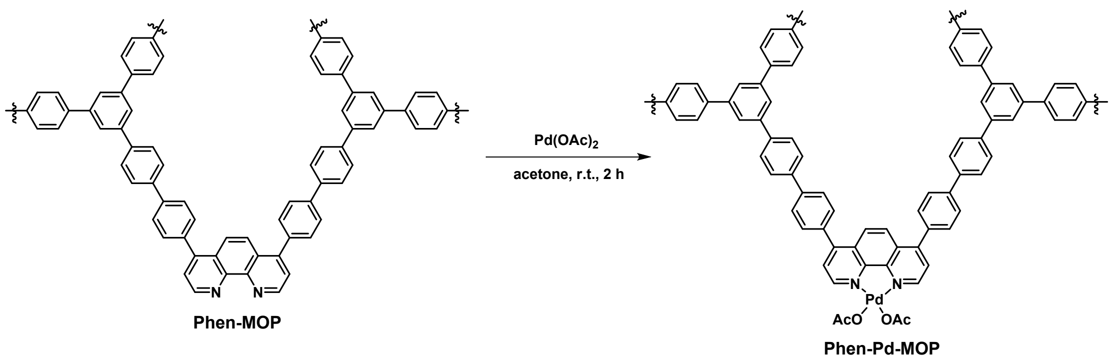

Palladium acetate (45 mg, 0.2 mmol) was dissolved in 10 mL of acetone, and then **Phen-MOP** (100 mg, 0.08 mmol) was added. The mixture was kept stirring for 2 h at room temperature. After the reaction was complete, the resulting solid was filtered and washed thoroughly with acetone (10 mL × 5), distilled water (10 mL × 5) and MeOH (10 mL × 5), then dried at 80 ^{o}C under vacuum for 12 h to yield **Phen-Pd-MOP** as a brown powder (110 mg). The Pd content in **Phen-Pd-MOP** was 10.60 % as determined by ICP.

C. N_2 adsorption-desorption isotherms of **Phen-Pd-MOP** and the recycled **Phen-Pd-MOP**

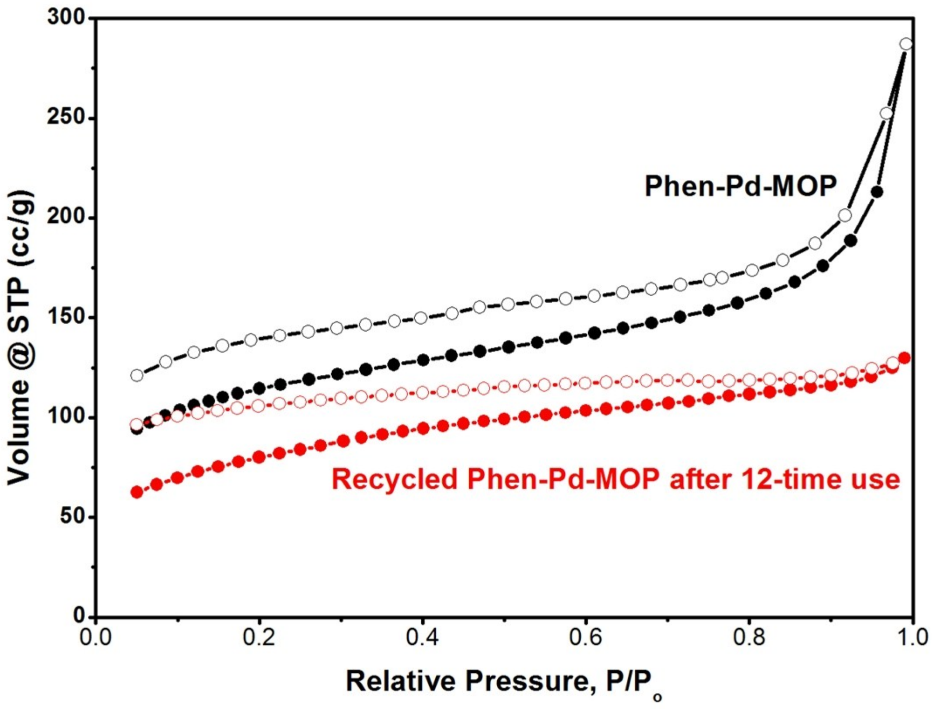

Fig. S1. N_2 adsorption-desorption isotherms of **Phen-Pd-MOP** and the recycled **Phen-Pd-MOP** after 12-time catalytic reactions. the BET surface area of **Phen-Pd-MOP** decrease to 403 m^2/g, which is lower than **Phen-MOP** due to the $Pd(OAc)_2$ is handing in the nanopores volume. We also found that the BET surface area of **Phen-Pd-MOP** had decreased to 272 m^2/g after the 12th recycled used, which could be due to the partial blocking of the polymeric nanopores by substrates and products.

D. N 1s XPS spectra for **Phen-MOP** and **Phen-Pd-MOP**

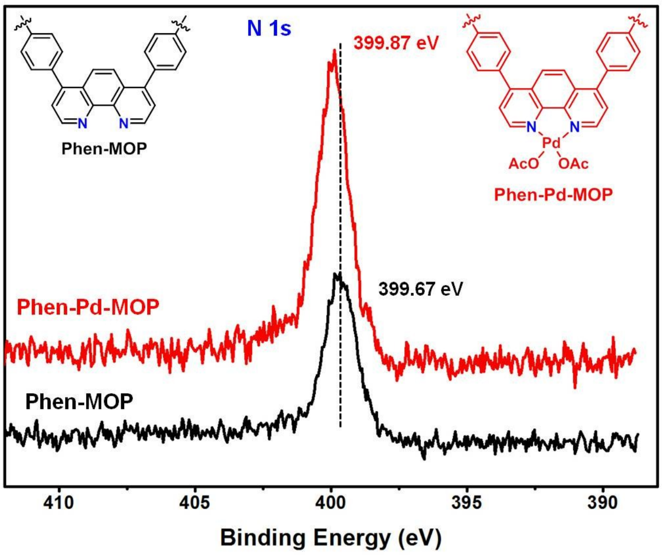

Fig. S2. N 1s XPS spectra for **Phen-MOP** and **Phen-Pd-MOP**

We also investigated the chemical states of N element in Phen-MOP and Phen-Pd-MOP frameworks by XPS spectrum. In comparison with Phen-MOP and Phen-Pd-MOP, the binding energy of N 1s in Phen-Pd-MOP was shifted from 399.67 to 399.87 eV, which further indicated that the $Pd(OAc)_2$ has strong coordinated with the *N,N*-bidentate ligand in Phen-MOP.

E. Powder X-Ray diffraction for **Phen-MOP**

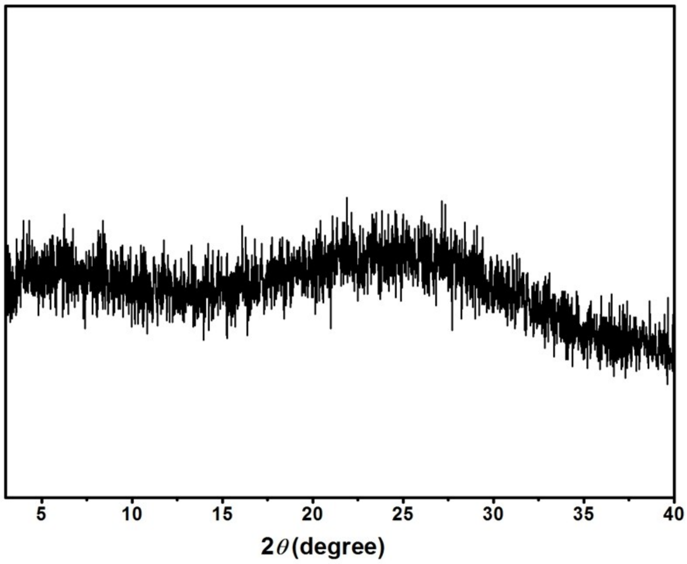

Figure S3. Powder X-ray diffraction pattern of **Phen-MOP**. No intensive diffraction peaks were observable.

F. Screening conditions for the Suzuki-Miyaura coupling reaction

Table S1. Screening conditions for the Suzuki-Miyaura coupling reaction.[a]

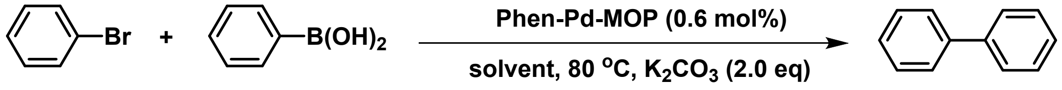

**Entry**	**Solvent**	**Time (h)**	**Yield (%)[b]**	**TON[e]**
1	EtOAc	4	30	50
2	Toluene	4	55	92
3	H_2O	4	75	125
4	CH_2Cl_2	4	60	100
5	THF	6	35	58
6	DMF	6	62	103
7	EtOH	1	95	158
8	MeOH	1	99	165
9	EtOH/ H_2O (1:1)	0.5	99	165
10[c]	EtOH/ H_2O (1:1)	4	0	0
11[d]	EtOH/ H_2O (1:1)	4	0	0

[a] General condition: phenylboronic acid (0.5 mmol), bromobenzene (0.75 mmol), K_2CO_3 (1.0 mmol), and **Phen-Pd-MOP** (0.6 mol%), solvent (1.0 mL), 80 °C.
[b] Isolated yield after silica gel column chromatography.
[c] In the absence of **Phen-Pd-MOP**.
[d] **Phen-MOP** as the catalyst.
[e] TON = (moles of product)/(moles of Pd in the catalyst).

G. General procedure for the Suzuki-Miyaura coupling reaction[3]

In a typical run of catalytic activity test of **Phen-Pd-MOP,** aryl halide (0.5 mmol), phenylboronic acid (91.5 mg, 0.75 mmol, 1.5 eq), K_2CO_3 (138 mg, 1.0 mmol, 2.0 eq), and **Phen-Pd-MOP** (3.0 mg, 0.6 mol%) were added to 1.0 mL of 1.0/1.0 EtOH-H_2O (v/v) mixture. The reaction mixture was stirred at 80 ^oC under ambient atmosphere. After the reaction was completed (monitored by TLC), the mixture was centrifuged and the solid was washed with EtOH (1 x 5 mL) and EtOAc (3 x 5 mL). The combined organic phase was washed with water to remove K_2CO_3 residue. After the evaporation of the solvent under vacuum, the residure was purified by flash column chromatography with petroleum ether or petroleum ether/EtOAc = 10:1 as the eluent.

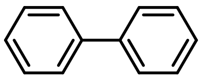

Prepared according to the typical procedure with bromobenzene (or iodobenzene) and phenylboronic acid. The product was collected as clear oil after silica gel chromatography with petroleum ether (77.0 mg, 99%). ^1H NMR (400 MHz, $CDCl_3$): δ 7.65–7.58 (m, 4H), 7.46 (t, J = 7.6 Hz, 4H), 7.37 (t, J = 7.3 Hz, 2H). ^{13}C NMR (100 MHz, $CDCl_3$): δ 141.2, 128.7, 127.2, 127.1.

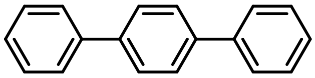

Prepared according to the typical procedure with 4-bromobiphenyl and phenylboronic acid. The product was collected as a white solid after silica gel chromatography with petroleum ether (109.8 mg, 95%). ^1H NMR (400 MHz, $CDCl_3$): δ 7.73–7.63 (m, 8H), 7.48 (t, J = 7.6 Hz, 4H), 7.38 (t, J = 7.4 Hz, 2H). ^{13}C NMR (100 MHz, $CDCl_3$): δ 140.7, 140.1, 128.8, 127.5, 127.3, 127.0.

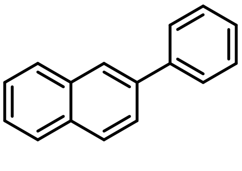

Prepared according to the typical procedure with 2-bromonaphthalene and

phenylboronic acid. The product was collected as a white solid after silica gel chromatography with petroleum ether (99.1 mg, 97%). ^{1}H NMR (400 MHz, $CDCl_3$): δ 8.06 (d, J = 1.1 Hz, 1H), 7.98–7.85 (m, 3H), 7.75 (tt, J = 3.1, 1.9 Hz, 3H), 7.55–7.46 (m, 4H), 7.42–7.38 (m, 1H). ^{13}C NMR (100 MHz, $CDCl_3$): δ 141.1, 138.6, 133.7, 132.6, 128.8, 128.4, 128.2, 127.6, 127.4, 127.3, 126.3, 125.9, 125.8, 125.6.

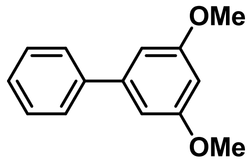

Prepared according to the typical procedure with 1-bromo-3,5-dimethoxybenzene and phenylboronic acid. The product was collected as a pale yellow solid after silica gel chromatography with petroleum ether/EtOAc = 10:1 (101.1 mg, 94%). ^{1}H NMR (400 MHz, $CDCl_3$): δ 7.60 (d, J = 7.5 Hz, 2H), 7.49–7.41 (m, 2H), 7.36 (dd, J = 15.4, 8.1 Hz, 1H), 6.76 (d, J = 2.2 Hz, 2H), 6.50 (t, J = 2.2 Hz, 1H), 3.87 (s, 6H). ^{13}C NMR (100 MHz, $CDCl_3$): δ 161.1, 143.5, 141.2, 128.7, 127.5, 127.2, 105.5, 99.3, 55.4.

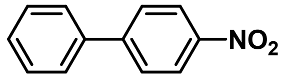

Prepared according to the typical procedure with *p*-nitrobromobenzene and phenylboronic acid. The product was collected as a pale yellow solid after silica gel chromatography with petroleum ether/EtOAc = 10:1 (92.8 mg, 93%). ^{1}H NMR (400 MHz, $CDCl_3$): δ 8.31 (d, J = 8.8 Hz, 2H), 7.75 (d, J = 8.8 Hz, 2H), 7.64 (d, J = 7.4 Hz, 2H), 7.54–7.37 (m, 3H).. ^{13}C NMR (100 MHz, $CDCl_3$): δ 147.6, 147.1, 138.8, 129.1, 128.9, 127.4, 124.1.

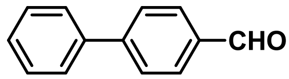

Prepared according to the typical procedure with 4-bromobenzaldehyde and phenylboronic acid. The product was collected as a pale yellow solid after silica gel chromatography with petroleum ether/EtOAc = 10:1 (83.6 mg, 92%). ^{1}H NMR (400 MHz, $CDCl_3$): δ 10.08 (s, 1H), 8.01–7.92 (m, 2H), 7.81–7.74 (m, 2H), 7.69–7.62 (m,

2H), 7.53–7.46 (m, 2H), 7.45–7.41 (m, 1H). ^{13}C NMR (100 MHz, $CDCl_3$): δ 191.9, 147.2, 139.7, 135.2, 130.2, 129.0, 128.5, 127.9, 127.3.

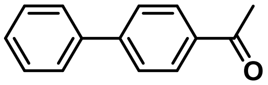

Prepared according to the typical procedure with *p*-bromoacetophenones and phenylboronic acid. The product was collected as a pale yellow solid after silica gel chromatography with petroleum ether/EtOAc = 10:1 (95.0 mg, 97%). ^{1}H NMR (400 MHz, $CDCl_3$): δ 8.09–8.01 (m, 2H), 7.70 (d, J = 8.4 Hz, 2H), 7.67–7.61 (m, 2H), 7.49 (dd, J = 10.2, 4.7 Hz, 2H), 7.45–7.38 (m, 1H), 2.65 (s, 3H). ^{13}C NMR (100 MHz, $CDCl_3$): δ 197.7, 145.8, 139.9, 135.8, 128.9, 128.8, 128.2, 127.2, 127.1, 26.6.

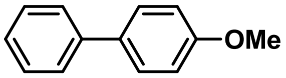

Prepared according to the typical procedure with 4-iodoanisole and phenylboronic acid. The product was collected as a white solid after silica gel chromatography with petroleum ether (90.2 mg, 98%). ^{1}H NMR (400 MHz, $CDCl_3$): δ 7.61–7.52 (m, 4H), 7.44 (dd, J = 10.4, 4.9 Hz, 2H), 7.37–7.28 (m, 1H), 7.06–6.94 (m, 2H), 3.87 (s, 3H). ^{13}C NMR (100 MHz, $CDCl_3$): δ 159.1, 140.8, 133.8, 128.7, 128.1, 126.7, 126.6, 114.2, 55.3.

H. Recyclability of **Phen-Pd-MOP** catalyst for the Suzuki-Miyaura

coupling reaction

The recycling experiment was performed by recovering the **Phen-Pd-MOP** catalyst using the centrifugation method. The recovered **Phen-Pd-MOP** catalyst was washed with EtOAc to remove the residual product and simply dried before resuse. We chose the Suzuki-Miyaura coupling reaction of bromobenzene to phenylboronic acid to investigate the recyclability of **Phen-Pd-MOP** catalyst, and the results are summarized in Table S2.

Table S2. Recycling of **Phen-Pd-MOP** catalyst for the Suzuki-Miyaura coupling reaction. [a]

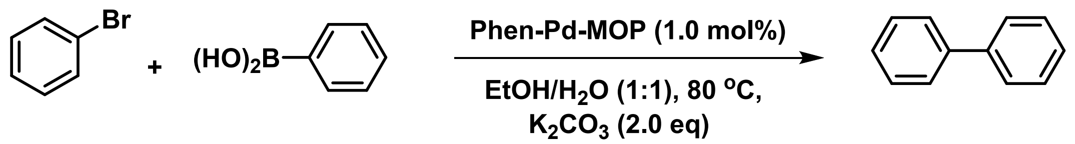

Cycle	Time (h)	Yield (%) [b]	Cycle	Time (h)	Yield (%) [b]
1	0.5	99	7	1	98
2	0.5	99	8	1	97
3	0.5	98	9	1	97
4	1	98	10	2	99
5	1	98	11	2	98
6	1	99	12	2	98

[a] General condition: phenylboronic acid (0.5 mmol), bromobenzene (0.75 mmol), K_2CO_3 (1.0 mmol), and **Phen-Pd-MOP** (0.005 mmol), EtOH/H_2O (0.5/0.5, 1.0 mL), 80 °C.
[b] Isolated yield after silica gel column chromatography.

I. Screening conditions for the Heck coupling reaction[4]

Table S3. Screening conditions for the Heck coupling reaction of iodobenzene with methyl acrylate in the presence of **Phen-Pd-MOP**.[a]

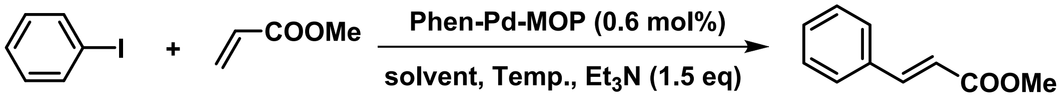

Entry	Solvent	Temp. (°C)	Time (h)	Yield (%)[b]	TON[e]
1	DMF	130	1	99	165
2	Toluene	130	3	55	92
3	THF	130	3	33	55
4	Acetone	130	3	80	133
5	H_2O	130	3	46	77
6	CH_2Cl_2	130	3	40	67
7	EtOH	130	3	75	125
8	EtOAc	130	3.5	82	137
9	DMF/ H_2O	130	3	88	147
10	DMF	100	6	75	125
11[c]	DMF	130	12	0	0
12[d]	DMF	130	12	0	0

[a] General condition: iodobenzene (0.5 mmol), methyl acrylate (0.75 mmol), Et_3N (0.75 mmol), and **Phen-Pd-MOP** (0.6 mol%), solvent (1.0 mL), temperature.
[b] Isolated yield after silica gel column chromatography.
[c] In the absence of **Phen-Pd-MOP**.
[d] **Phen-MOP** as the catalyst.
[e] TON = (moles of product)/(moles of Pd in the catalyst).

J. General procedure for the Heck coupling reaction

In a typical run of catalytic activity test of **Phen-Pd-MOP** for the Heck coupling reaction**,** aryl halides (0.5 mmol), methyl acrylate (64.5 mg, 0.75 mmol, 1.5 eq), Et_3N (0.75 mmol, 1.5 eq), and **Phen-Pd-MOP** (3.0 mg, 0.6 mol%) were added to 1.0 mL DMF. The reaction mixture was stirred at 130 ^{o}C under N_2 atmosphere. After the reaction was completed (monitored by TLC), the mixture was cooled down to room temperature, centrifuged and the **Phen-Pd-MOP** catalyst was washed with EtOAc (3 x 5 mL). The combined organic phase was washed with water and dried over anhydrous Na_2SO_4. After the evaporation of the solvent under vacuum, the residue was purified by flash column chromatography with petroleum ether/EtOAc (6:1 to 20:1) as the eluent.

Table 2, Entry 1 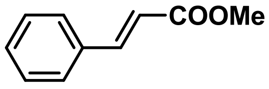

Prepared according to the typical procedure with iodobenzene and methyl acrylate. The product was collected as pale yellow oil after silica gel chromatography with petroleum ether/EtOAc = 20:1. ^{1}H NMR (400 MHz, $CDCl_3$) δ 7.71 (d, J = 16.0 Hz, 1H), 7.55–7.53 (m, 2H), 7.40–7.39 (m, 3H), 6.45 (d, J = 16.0 Hz, 1H), 3.82 (s, 3H). ^{13}C NMR (100 MHz, $CDCl_3$): δ 167.4, 144.9, 134.4, 130.3, 128.9, 128.1, 117.8, 51.7.

Table 2, Entry 2 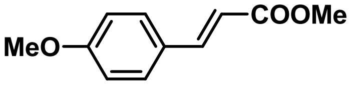

Prepared according to the typical procedure with 4-iodoanisole and methyl acrylate. The product was collected as white solid after silica gel chromatography with petroleum ether/EtOAc = 10:1. ^{1}H NMR (400 MHz, $CDCl_3$) δ 7.65 (d, J = 16.0 Hz, 1H), 7.47 (d, J = 8.7 Hz, 2H), 6.90 (d, J = 8.7 Hz, 2H), 6.31 (d, J = 16.0 Hz, 1H), 3.83 (s, 3H), 3.79 (s, 3H). ^{13}C NMR (100 MHz, $CDCl_3$) δ 167.7, 161.3, 144.4, 129.6, 127.0, 115.2, 114.2, 55.3, 51.5.

Table 2, Entry 3 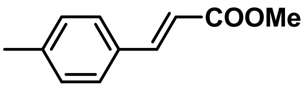

Prepared according to the typical procedure with 4-iodotoluene and methyl acrylate. The product was collected as white solid after silica gel chromatography with petroleum ether/EtOAc = 20:1. ^{1}H NMR (400 MHz, $CDCl_3$) δ 7.68 (d, *J* = 16.0 Hz, 1H), 7.43 (d, *J* = 8.1 Hz, 2H), 7.19 (d, *J* = 8.0 Hz, 2H), 6.41 (d, *J* = 16.0 Hz, 1H), 3.81 (s, 3H), 2.38 (s, 3H). ^{13}C NMR (100 MHz, $CDCl_3$) δ 167.5, 144.8, 140.7, 131.6, 129.6, 128.0, 116.7, 51.5, 21.4.

Table 2, Entry 4 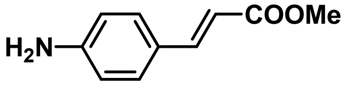

Prepared according to the typical procedure with 4-iodoaniline and methyl acrylate. The product was collected as yellow oil after silica gel chromatography with petroleum ether/EtOAc = 6:1. ^{1}H NMR (400 MHz, $CDCl_3$) δ 7.62–7.57 (m, 1H), 7.32–7.30 (m, 2H), 6.61 (dd, *J* = 10.8, 9.4 Hz, 2H), 6.22 (dt, *J* = 20.7, 10.3 Hz, 1H), 3.76 (d, *J* = 1.0 Hz,3H), 2.93–2.86 (m, 2H). ^{13}C NMR (100 MHz, $CDCl_3$) δ 168.1, 162.5, 148.9, 145.1, 129.7, 114.6, 112.8, 51.3.

Table 2, Entry 5 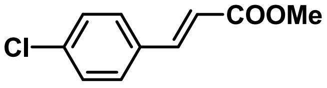

Prepared according to the typical procedure with 1-chloro-4-iodobenzene and methyl acrylate. The product was collected as white solid after silica gel chromatography with petroleum ether/EtOAc = 10:1. ^{1}H NMR (400 MHz, $CDCl_3$) δ 7.63 (d, *J* = 16.0 Hz, 1H), 7.45–7.43 (m, 2H), 7.36 –7.34 (m, 2H), 6.40 (dd, *J* = 16.0, 0.9 Hz, 1H), 3.80 (s, 3H). ^{13}C NMR (100 MHz, $CDCl_3$) δ 167.1, 143.3, 136.1, 132.8, 129.2, 129.1, 51.7.

Table 2, Entry 6 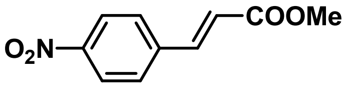

Prepared according to the typical procedure with 1-iodo-4-nitrobenzene and methyl acrylate. The product was collected as yellow solid after silica gel chromatography with petroleum ether/EtOAc = 6:1. ^{1}H NMR (400 MHz, $CDCl_3$) δ 8.25 (d, *J* = 8.6 Hz, 2H), 7.74–7.66 (m, 3H), 6.56 (d, *J* = 16.1 Hz, 1H), 3.84 (s, 3H).^{13}C NMR (100 MHz, $CDCl_3$) δ 166.4, 141.9, 140.5, 128.6, 128.3, 124.3, 122.1, 52.0.

Table 2, Entry 7 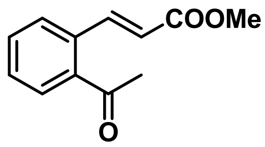

Prepared according to the typical procedure with 1-(2-iodophenyl)ethanone and methyl acrylate. The product was collected as yellow oil after silica gel chromatography with petroleum ether/EtOAc = 6:1. ^{1}H NMR (400 MHz, $CDCl_3$) δ 8.16 (d, *J* = 15.9 Hz, 1H), 7.75 (td, *J* = 7.4, 1.3 Hz, 1H), 7.55 – 7.52 (m, 1H), 7.51 – 7.45 (m, 2H), 6.29 (d, *J* = 15.9 Hz, 1H), 3.82 (s, 4H), 2.63 (s, 3H).

Table 2, Entry 8 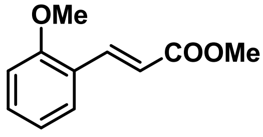

Prepared according to the typical procedure with 2-iodoanisole and methyl acrylate. The product was collected as yellow oil after silica gel chromatography with petroleum ether/EtOAc = 10:1. ^{1}H NMR (400 MHz, $CDCl_3$) δ 8.01 (d, *J* = 16.2 Hz, 1H), 7.52 (dd, *J* = 7.7, 1.5 Hz, 1H), 7.38–7.34 (m, 1H), 76.99 – 6.91 (m, 2H), 6.54 (d, *J* = 16.2 Hz, 1H), 3.90 (s, 3H), 3.81 (s, 3H). ^{13}C NMR (100 MHz, $CDCl_3$) δ 158.3, 140.3, 131.5, 128.9, 123.4, 120.7, 118.3, 111.1, 55.5, 51.6.

Table 2, Entry 9 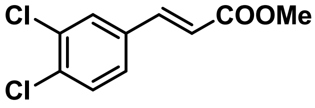

Prepared according to the typical procedure with 1,2-dichloro-4-iodobenzene and methyl acrylate. The product was collected as white solid after silica gel chromatography with petroleum ether/EtOAc = 10:1. ^{1}H NMR (400 MHz, $CDCl_3$) δ 7.54 (d, *J* = 16.0 Hz, 1H), 7.38–7.36 (m, 3H), 6.43 (d, *J* = 16.0 Hz, 1H), 3.82 (s, 3H). ^{13}C NMR (100 MHz, $CDCl_3$) δ 166.5, 141.7, 137.3, 135.5, 129.8, 126.2, 120.6, 51.9.

Table 2, Entry 11 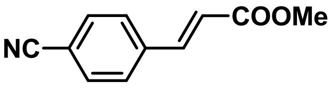

Prepared according to the typical procedure with 4-iodobenzonitrile and methyl acrylate. The product was collected as yellow solid after silica gel chromatography with petroleum ether/EtOAc = 6:1. ^{1}H NMR (400 MHz, $CDCl_3$) δ 7.71–7.66 (m, 3H),

7.62 (d, J = 8.3 Hz, 2H), 6.53 (d, J = 16.0 Hz, 1H), 3.84 (s, 3H). ^{13}C NMR (100 MHz, $CDCl_3$) δ 166.6, 142.4, 138.7, 132.7, 128.4, 121.4, 113.4, 52.0.

Table 2, Entry 13 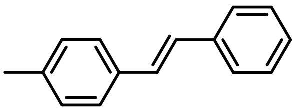

Prepared according to the typical procedure with 4-iodotoluene and styrene. The product was collected as white solid after silica gel chromatography with petroleum ether. ^{1}H NMR (400 MHz, $CDCl_3$) δ 7.50 (d, J = 7.4 Hz, 2H), 7.41 (d, J = 8.1 Hz, 2H), 7.34 (t, J = 7.6 Hz, 2H), 7.23 (t, J = 7.3 Hz, 1H), 7.16 (d, J = 7.9 Hz, 2H), 7.07 (d, J = 2.4 Hz, 2H), 2.36 (s, 3H). ^{13}C NMR (100 MHz, $CDCl_3$) δ 137.5, 134.6, 129.4, 128.6, 127.7, 127.4, 126.4, 126.3, 21.2.

K. Recyclability of **Phen-Pd-MOP** catalyst for the Heck coupling reaction

The recycling experiment was performed by recovering the **Phen-Pd-MOP** catalyst using the centrifugation method. The recovered **Phen-Pd-MOP** catalyst was washed with EtOAc to remove the residual product and simply dried before resuse. We chose the Heck coupling reaction of iodobenzene to methyl acrylate to investigate the recyclability of **Phen-Pd-MOP** catalyst, and the results are summarized in Table S4.

Table S4. Recycling of **Phen-Pd-MOP** catalyst for the Heck coupling reaction. [a]

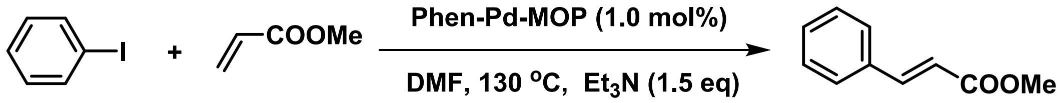

Cycle	Time (h)	Yield (%) [b]
1	1	99
2	1	98
3	1	98
4	2	99
5	2	97
6	2	98
7	4	98
8	4	98
9	4	97
10	4	97

[a] General condition: iodobenzene (0.5 mmol), methyl acrylate (0.75 mmol), Et_3N (0.75 mmol), and **Phen-Pd-MOP** (1.0 mol%), DMF (1.0 mL), 130 °C.

[b] Isolated yield after silica gel column chromatography.

References

[1] B. Li, Z. Guan, X. Yang, W. D. Wang, W. Wang, I. Hussain, K. Song, B. Tan and

T. Li, *J. Mater. Chem. A*, 2014, **2**, 11930.
[2] S.-Y. Ding, J. Gao, Q. Wang, Y. Zhang, W.-G. Song, C.-Y. Su and W. Wang, *J. Am. Chem. Soc.*, 2011, **133**, 19816.
[3] C.-A. Wang, Y.-F. Han, Y.-W. Li, K. Nie, X.-L. Cheng and J.-P. Zhang, *RSC Adv.*, 2016, **6**, 34866.
[4] H. Li, B. Xu, X. Liu, S. A, C. He, H. Xia and Y. Mu, *J. Mater. Chem. A*, 2013, **1**, 14108.

L. ^{1}H NMR and ^{13}C NMR spectra of the products

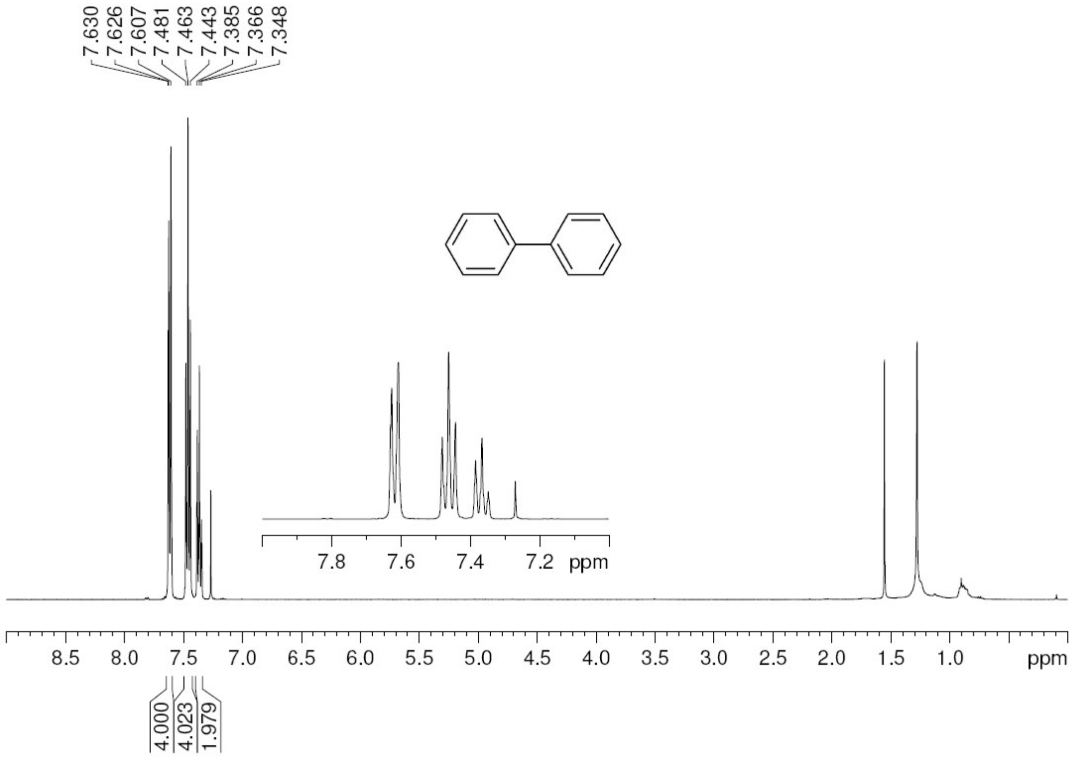
7.630
7.626
7.607
7.481
7.463
7.443
7.385
7.366
7.348
7.8
7.6
7.4
7.2
ppm
8.5
8.0
7.5
7.0
6.5
6.0
5.5
5.0
4.5
4.0
3.5
3.0
2.5
2.0
1.5
1.0
ppm
4.000
4.023
1.979

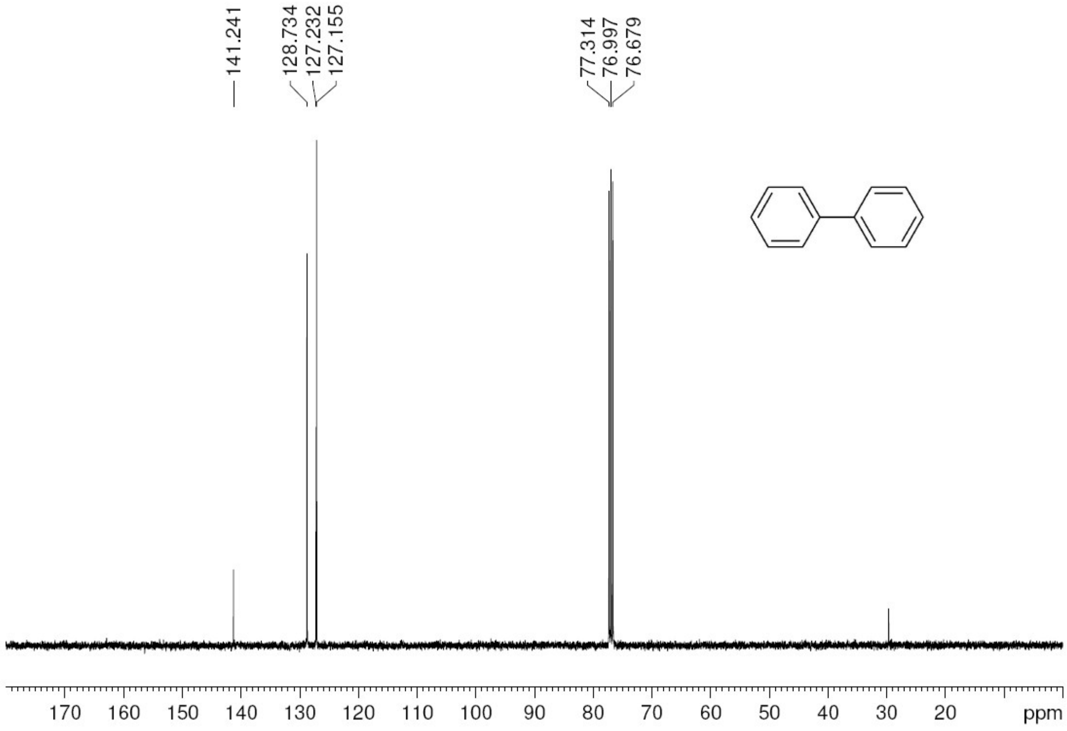
141.241
128.734
127.232
127.155
77.314
76.997
76.679
170
160
150
140
130
120
110
100
90
80
70
60
50
40
30
20
ppm

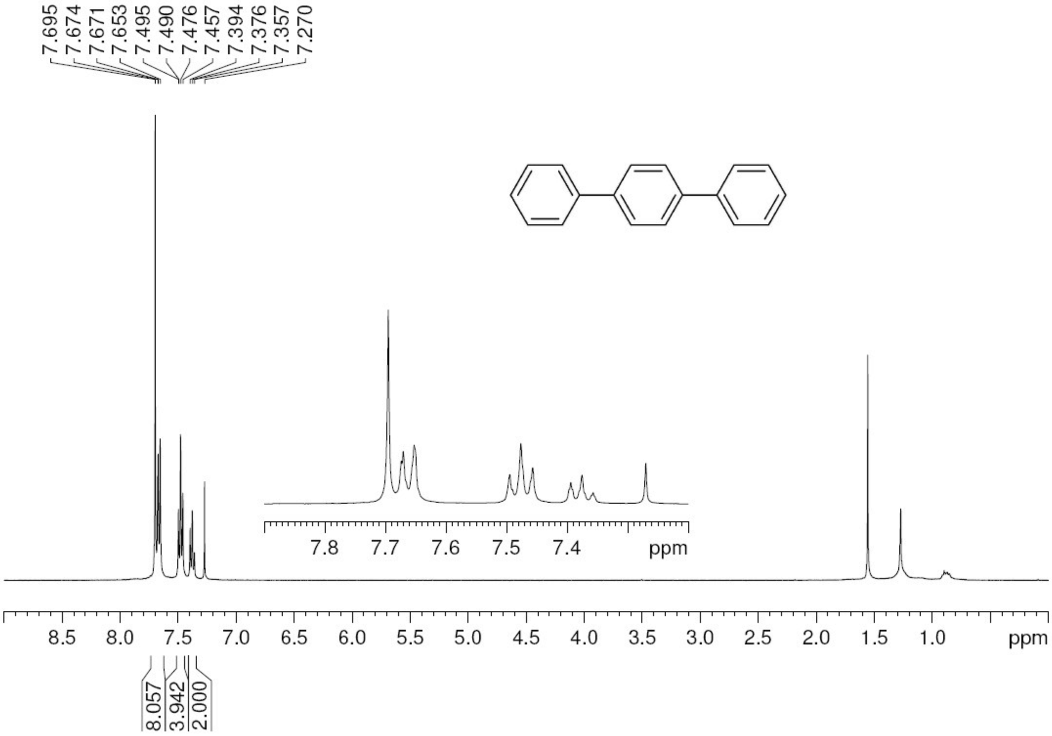
7.695
7.674
7.671
7.653
7.495
7.490
7.476
7.457
7.394
7.376
7.357
7.270
7.8
7.7
7.6
7.5
7.4
ppm
8.5
8.0
7.5
7.0
6.5
6.0
5.5
5.0
4.5
4.0
3.5
3.0
2.5
2.0
1.5
1.0
ppm
8.057
3.942
2.000

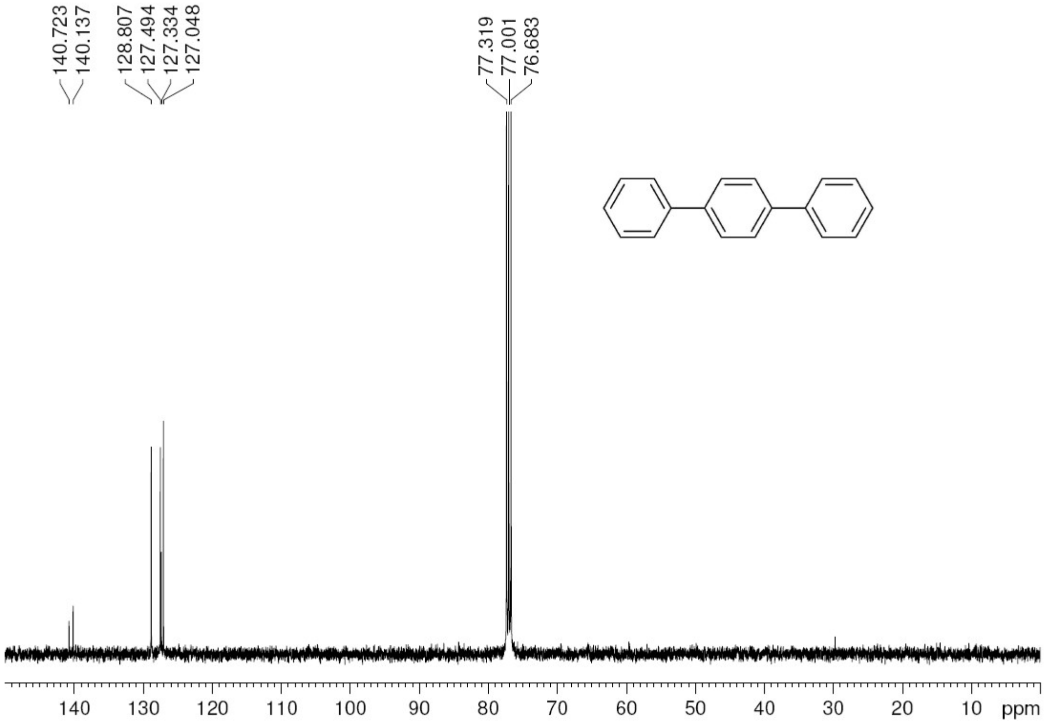
140.723
140.137
128.807
127.494
127.334
127.048
77.319
77.001
76.683
140
130
120
110
100
90
80
70
60
50
40
30
20
10
ppm

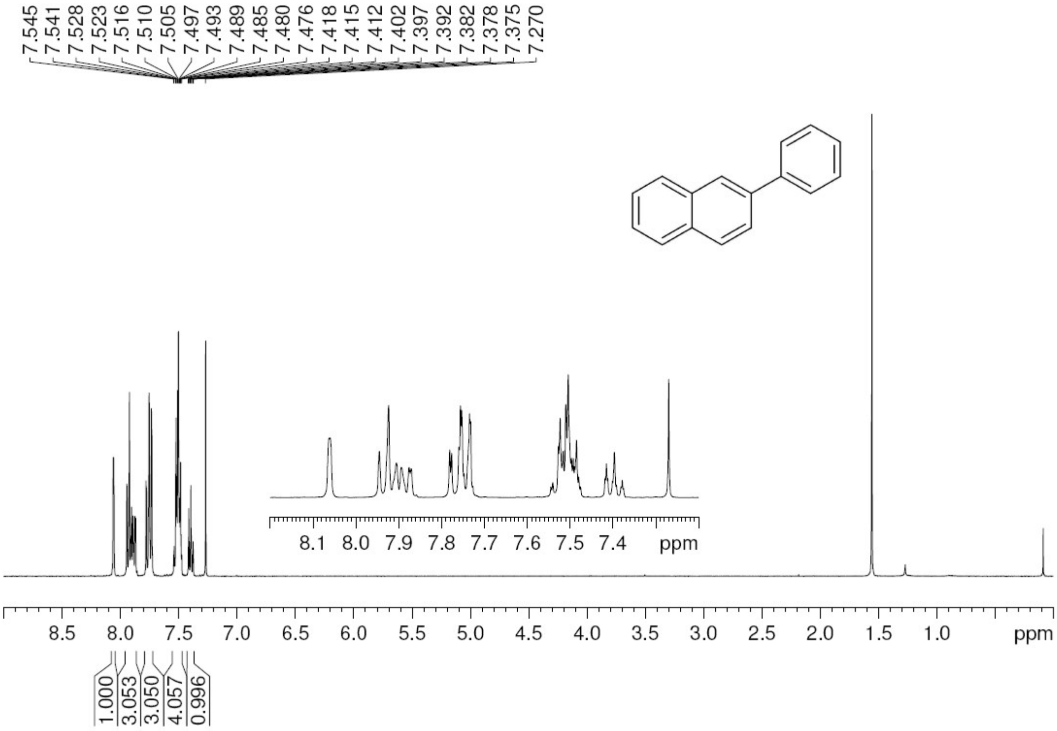
7.545 7.541 7.528 7.523 7.516 7.510 7.505 7.497 7.493 7.489 7.485 7.480 7.476 7.418 7.415 7.412 7.402 7.397 7.392 7.382 7.378 7.375 7.270
8.1 8.0 7.9 7.8 7.7 7.6 7.5 7.4 ppm
8.5 8.0 7.5 7.0 6.5 6.0 5.5 5.0 4.5 4.0 3.5 3.0 2.5 2.0 1.5 1.0 ppm
1.000 3.053 3.050 4.057 0.996

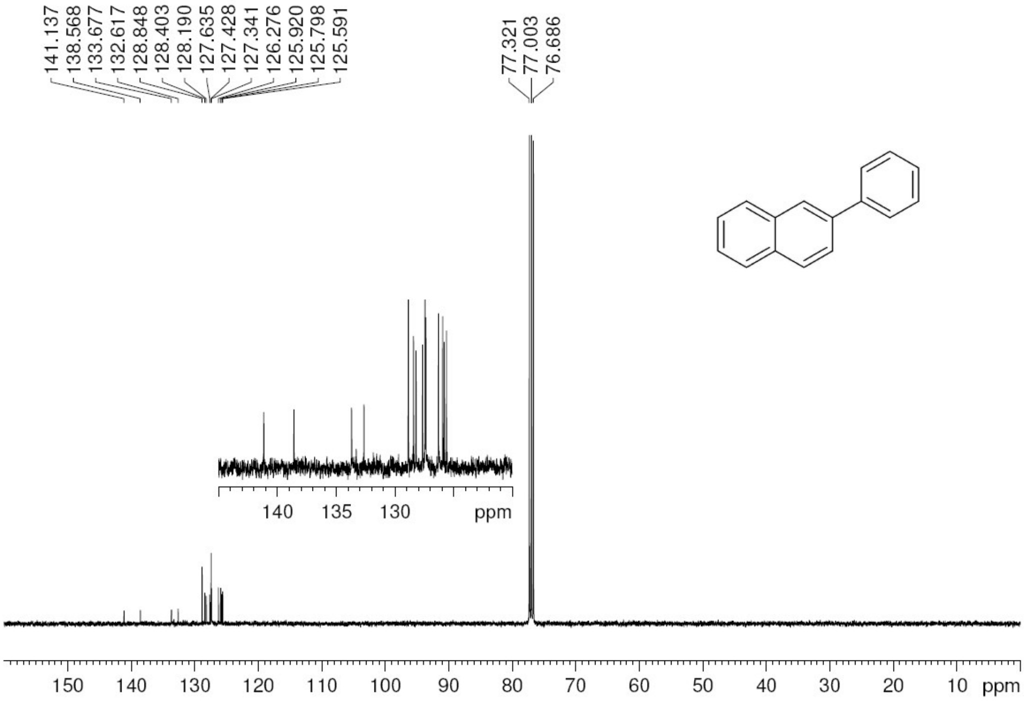
141.137 138.568 133.677 132.617 128.848 128.403 128.190 127.635 127.428 127.341 126.276 125.920 125.798 125.591
77.321 77.003 76.686
140 135 130 ppm
150 140 130 120 110 100 90 80 70 60 50 40 30 20 10 ppm

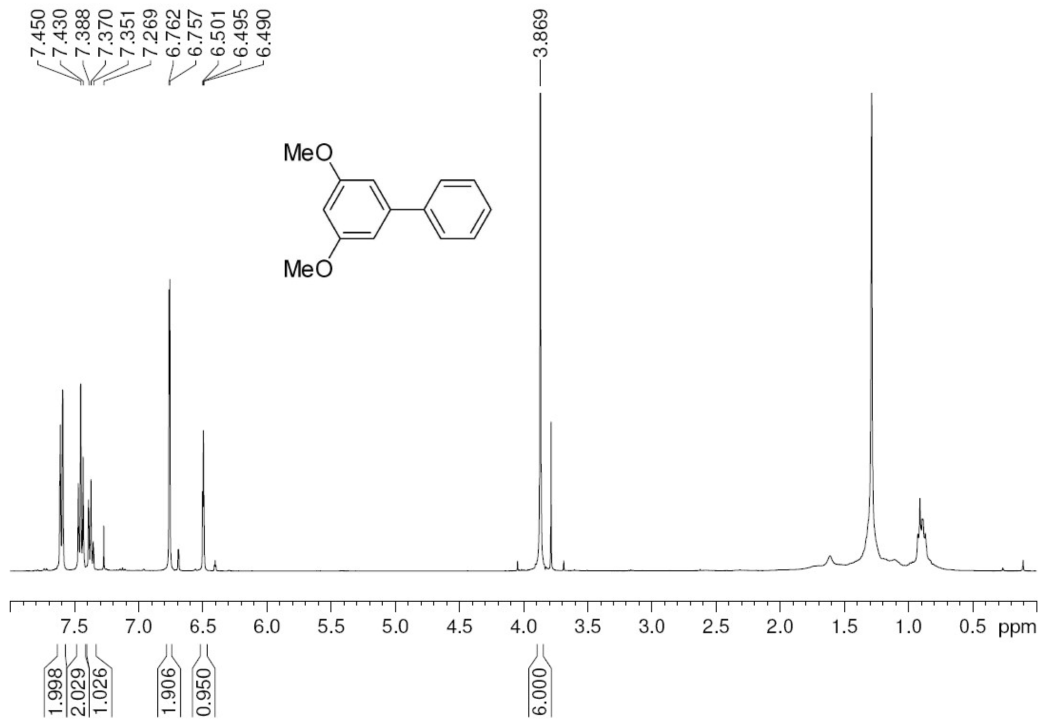
7.450
7.430
7.388
7.370
7.351
7.269
6.762
6.757
6.501
6.495
6.490
3.869
MeO
MeO
7.5 7.0 6.5 6.0 5.5 5.0 4.5 4.0 3.5 3.0 2.5 2.0 1.5 1.0 0.5 ppm
1.998
2.029
1.026
1.906
0.950
6.000

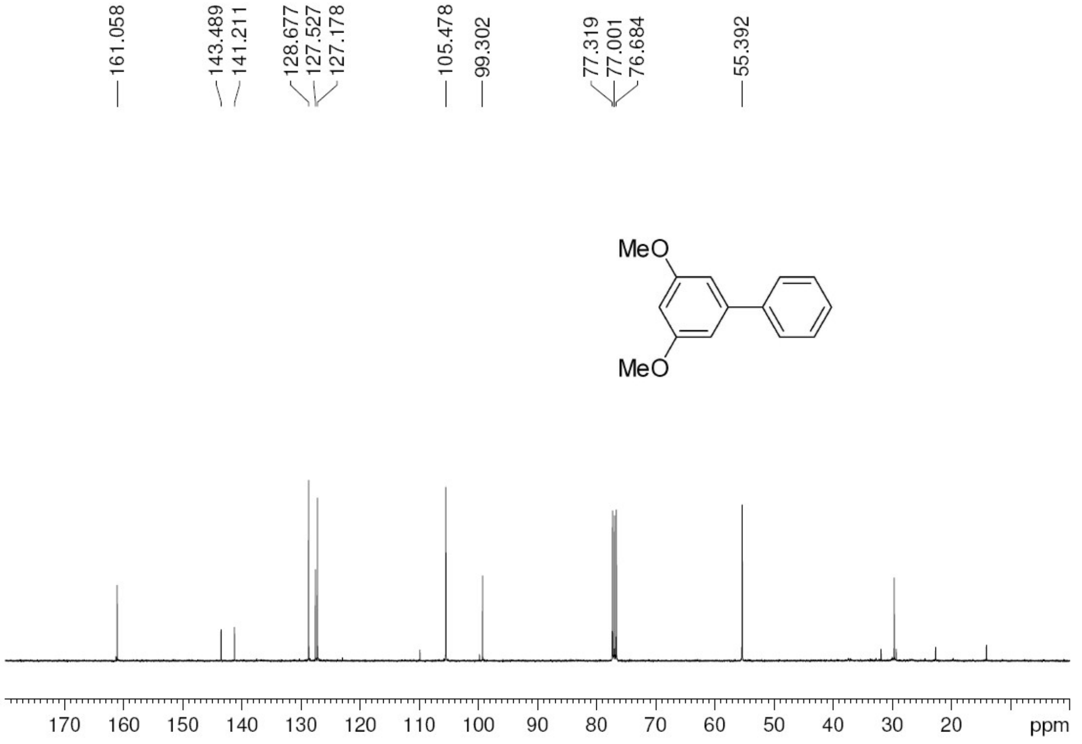
161.058
143.489
141.211
128.677
127.527
127.178
105.478
99.302
77.319
77.001
76.684
55.392
MeO
MeO
170 160 150 140 130 120 110 100 90 80 70 60 50 40 30 20 ppm

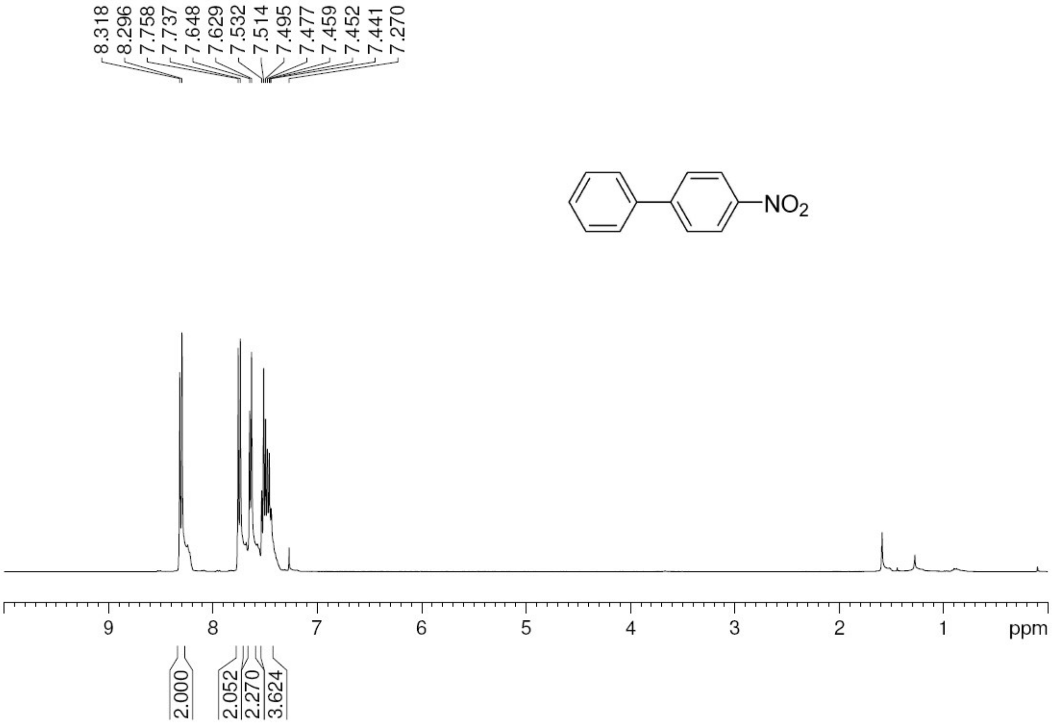
8.318
8.296
7.758
7.737
7.648
7.629
7.532
7.514
7.495
7.477
7.459
7.452
7.441
7.270
NO2
9
8
7
6
5
4
3
2
1
ppm
2.000
2.052
2.270
3.624

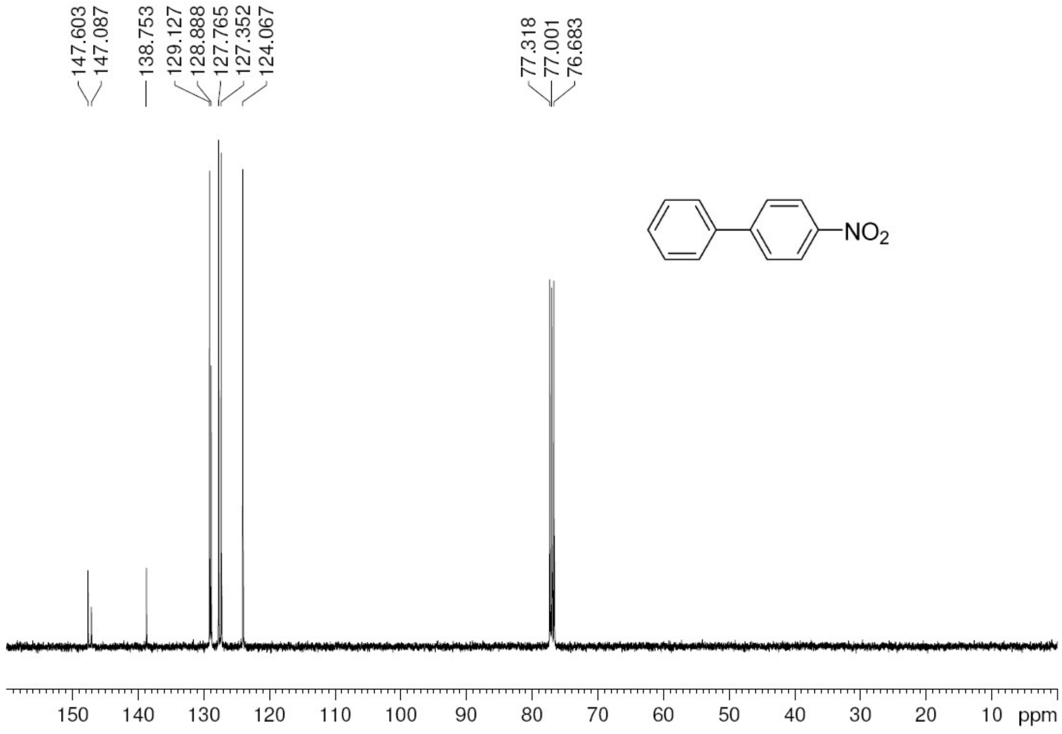
147.603
147.087
138.753
129.127
128.888
127.765
127.352
124.067
77.318
77.001
76.683
NO2
150
140
130
120
110
100
90
80
70
60
50
40
30
20
10
ppm

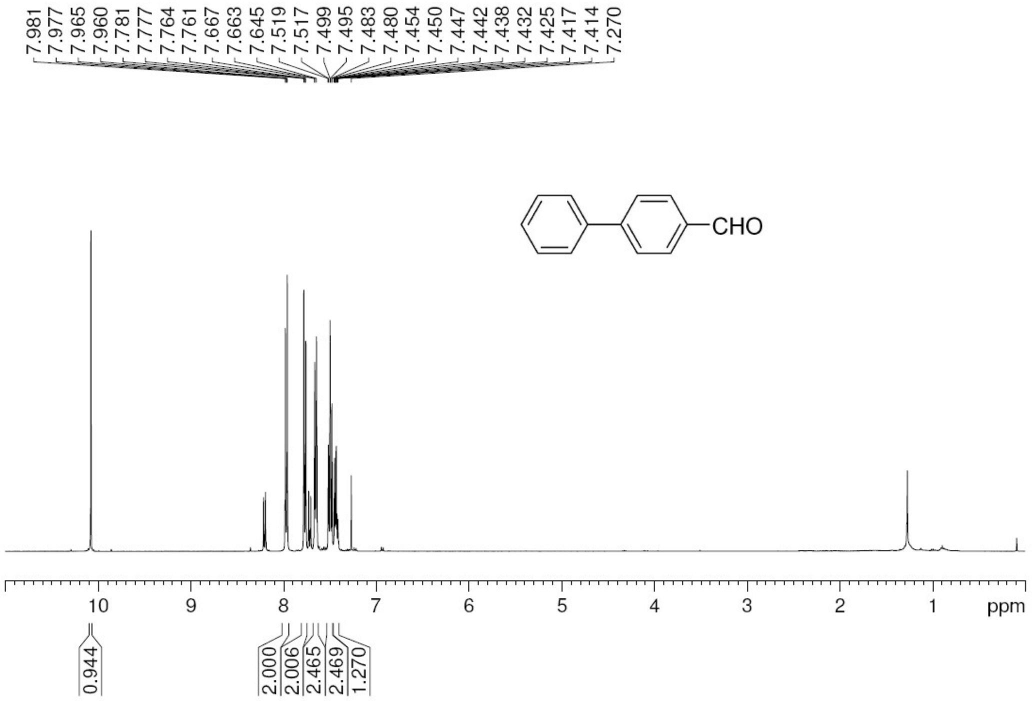
7.981
7.977
7.965
7.960
7.781
7.777
7.764
7.761
7.667
7.663
7.645
7.519
7.517
7.499
7.495
7.483
7.480
7.454
7.450
7.447
7.442
7.438
7.432
7.425
7.417
7.414
7.270
CHO
10
9
8
7
6
5
4
3
2
1
ppm
0.944
2.000
2.006
2.465
2.469
1.270

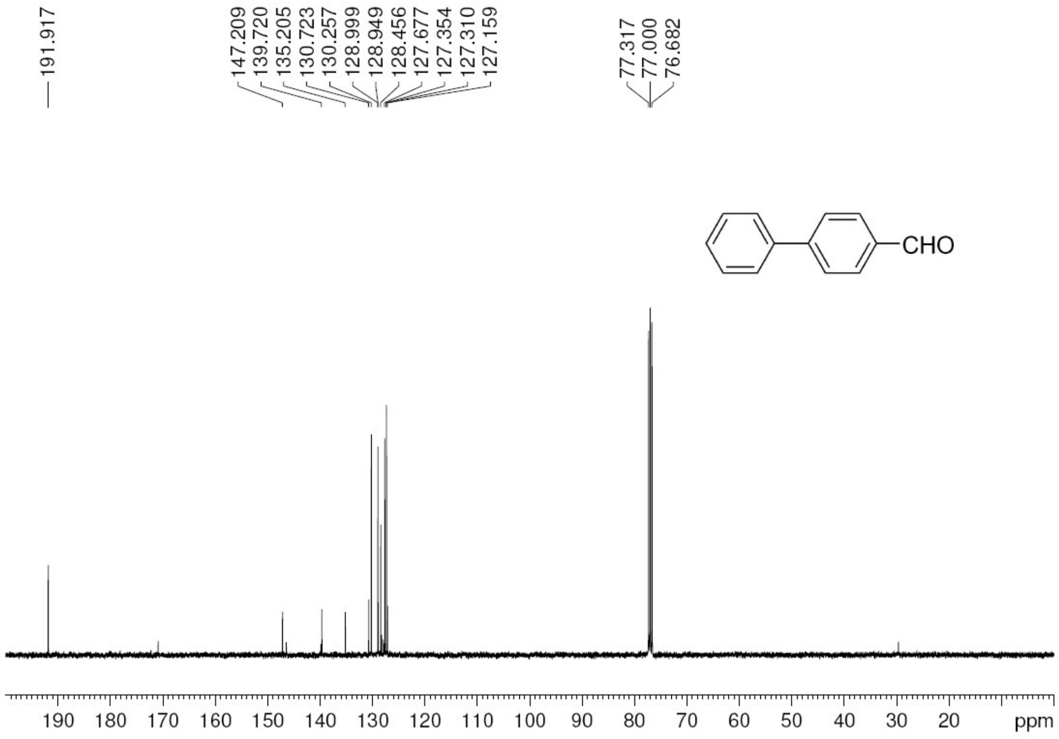
191.917
147.209
139.720
135.205
130.723
130.257
128.999
128.949
128.456
127.677
127.354
127.310
127.159
77.317
77.000
76.682
CHO
190
180
170
160
150
140
130
120
110
100
90
80
70
60
50
40
30
20
ppm

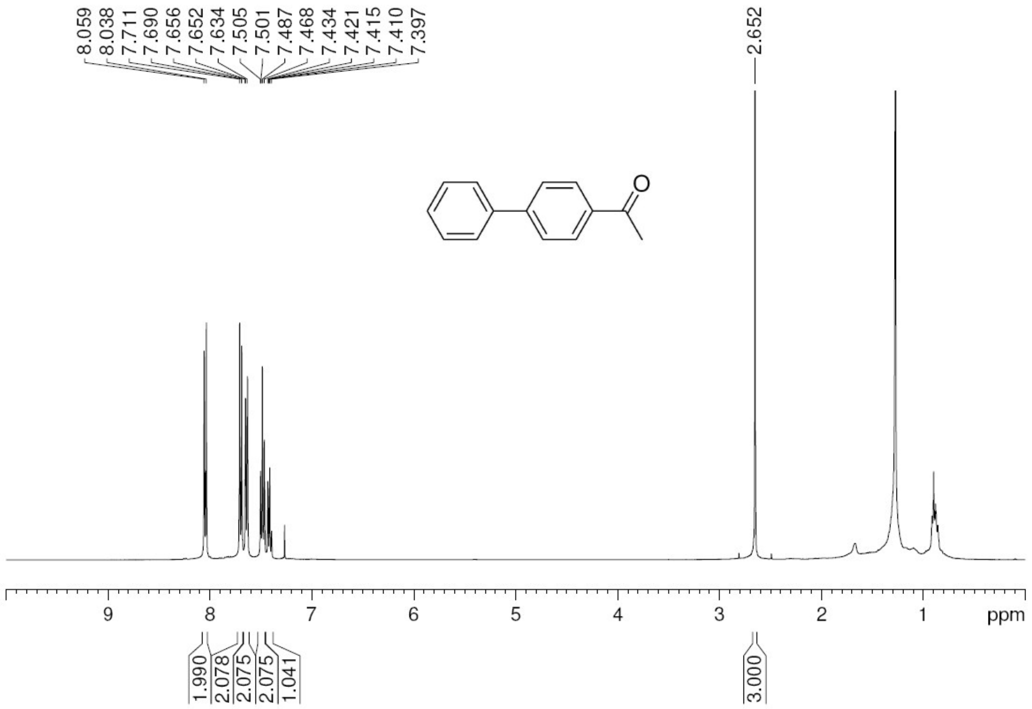
8.059
8.038
7.711
7.690
7.656
7.652
7.634
7.505
7.501
7.487
7.468
7.434
7.421
7.415
7.410
7.397
2.652
O
9
8
7
6
5
4
3
2
1
ppm
1.990
2.078
2.075
2.075
1.041
3.000

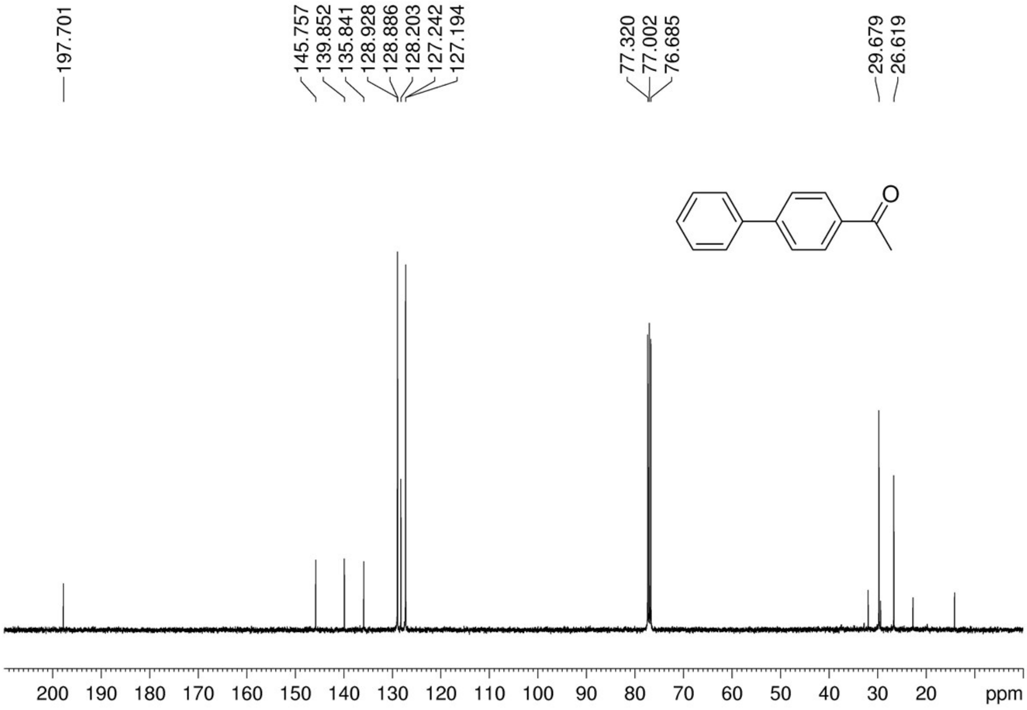
197.701
145.757
139.852
135.841
128.928
128.886
128.203
127.242
127.194
77.320
77.002
76.685
29.679
26.619
O
200
190
180
170
160
150
140
130
120
110
100
90
80
70
60
50
40
30
20
ppm

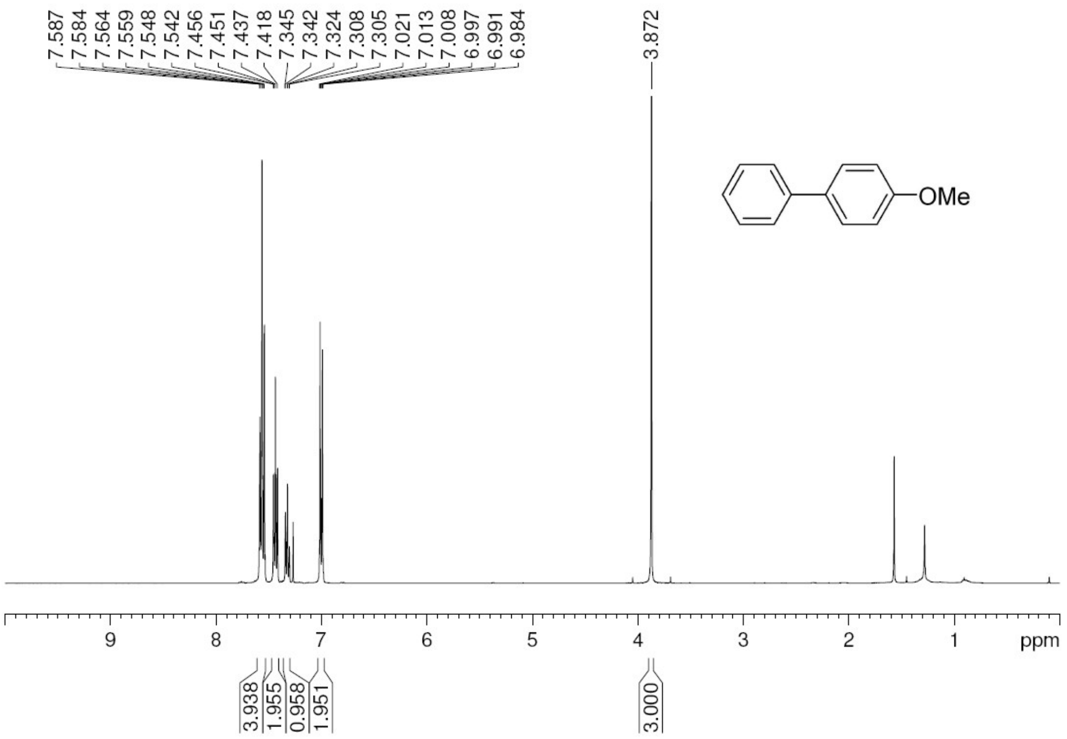

7.587
7.584
7.564
7.559
7.548
7.542
7.456
7.451
7.437
7.418
7.345
7.342
7.324
7.308
7.305
7.021
7.013
7.008
6.997
6.991
6.984
3.872
OMe
9 8 7 6 5 4 3 2 1 ppm
3.938
1.955
0.958
1.951
3.000

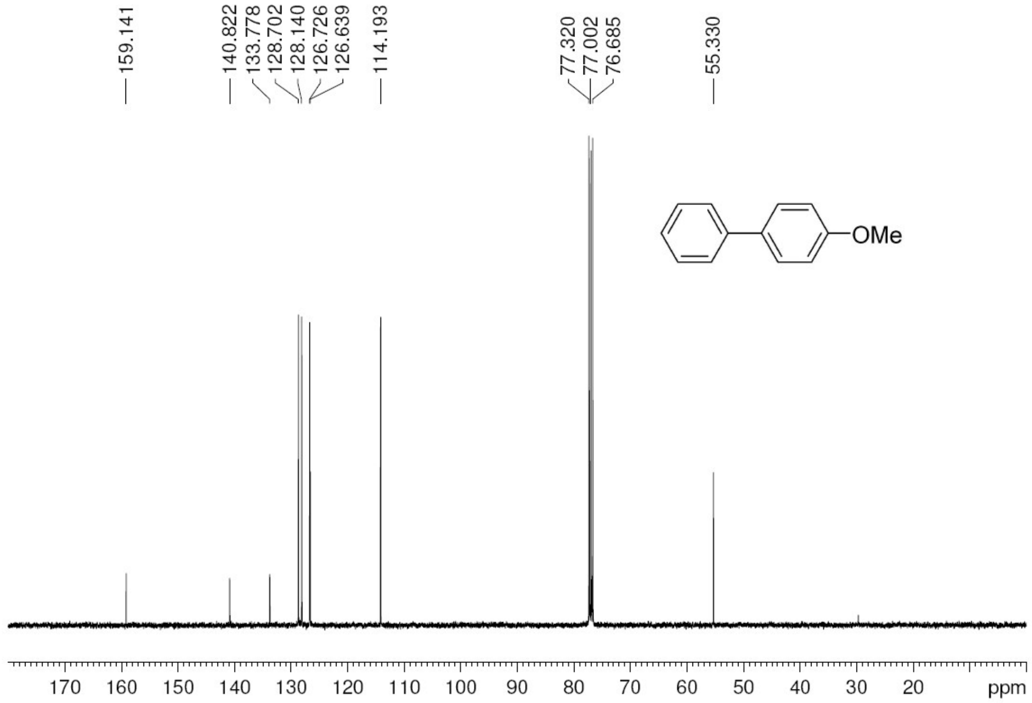

159.141
140.822
133.778
128.702
128.140
126.726
126.639
114.193
77.320
77.002
76.685
55.330
OMe
170 160 150 140 130 120 110 100 90 80 70 60 50 40 30 20 ppm

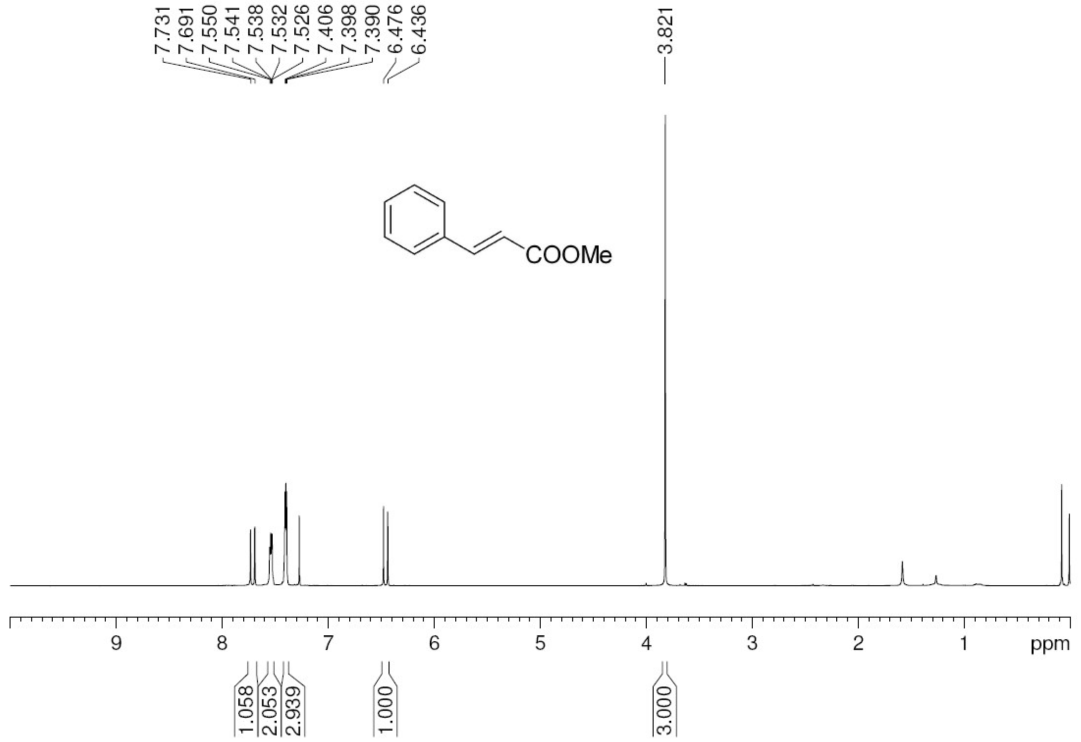
7.731
7.691
7.550
7.541
7.538
7.532
7.526
7.406
7.398
7.390
6.476
6.436
3.821
COOMe
9
8
7
6
5
4
3
2
1
ppm
1.058
2.053
2.939
1.000
3.000

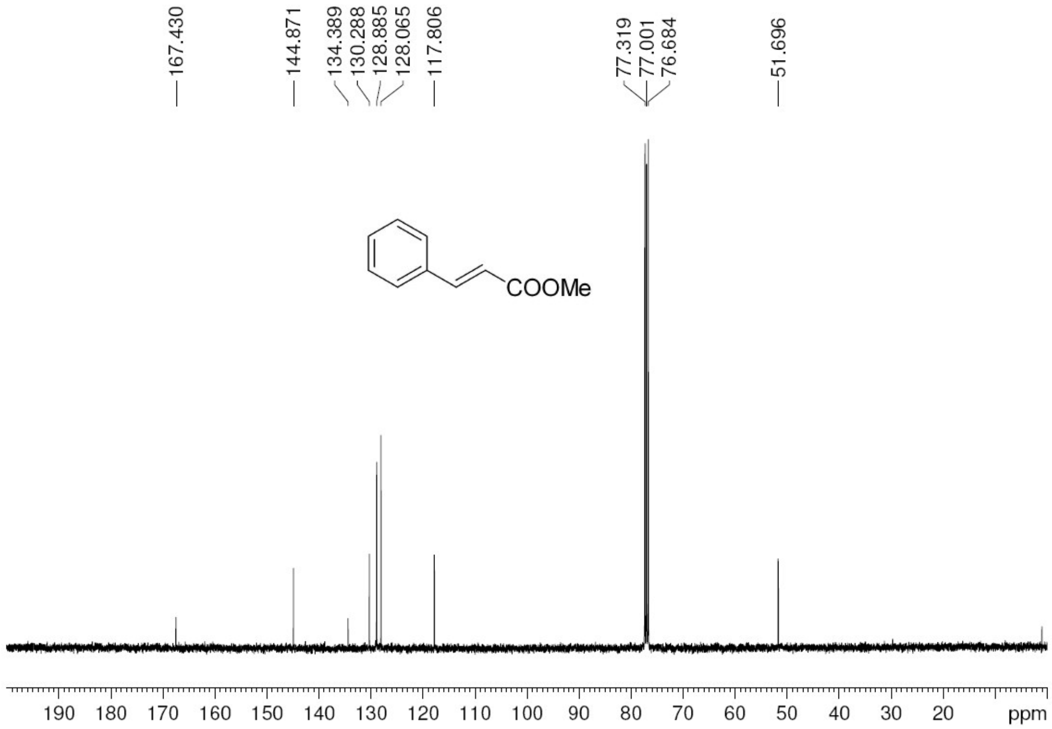
167.430
144.871
134.389
130.288
128.885
128.065
117.806
77.319
77.001
76.684
51.696
COOMe
190
180
170
160
150
140
130
120
110
100
90
80
70
60
50
40
30
20
ppm

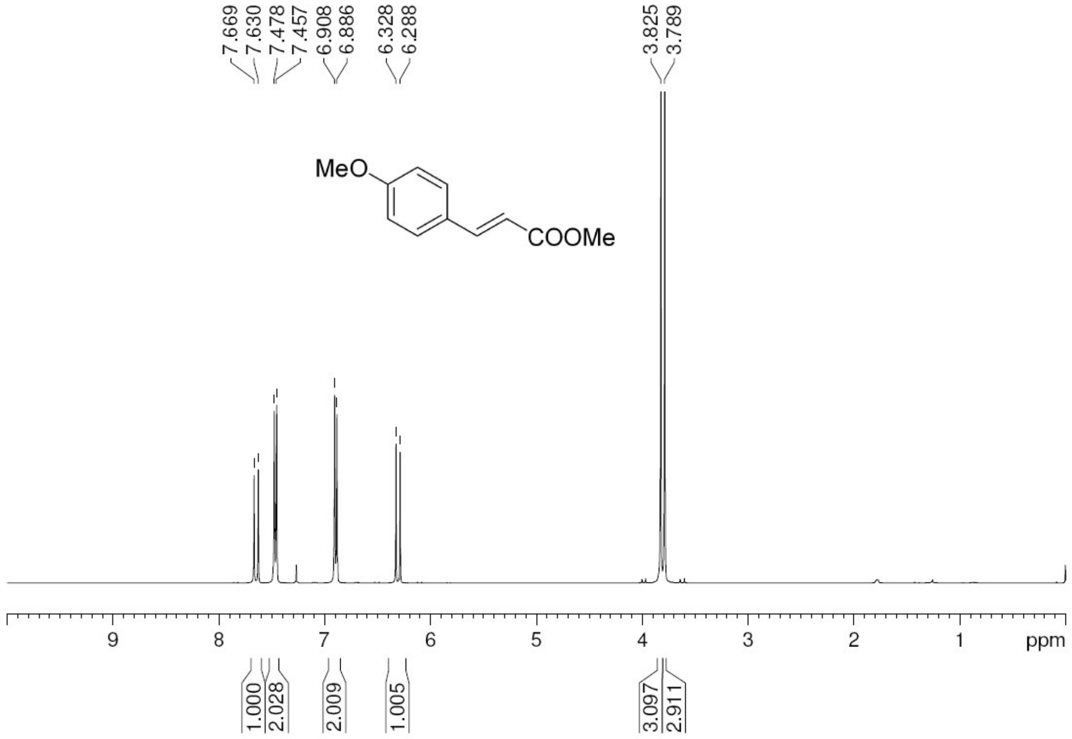
7.669
7.630
7.478
7.457
6.908
6.886
6.328
6.288
3.825
3.789
MeO
COOMe
9
8
7
6
5
4
3
2
1
ppm
1.000
2.028
2.009
1.005
3.097
2.911

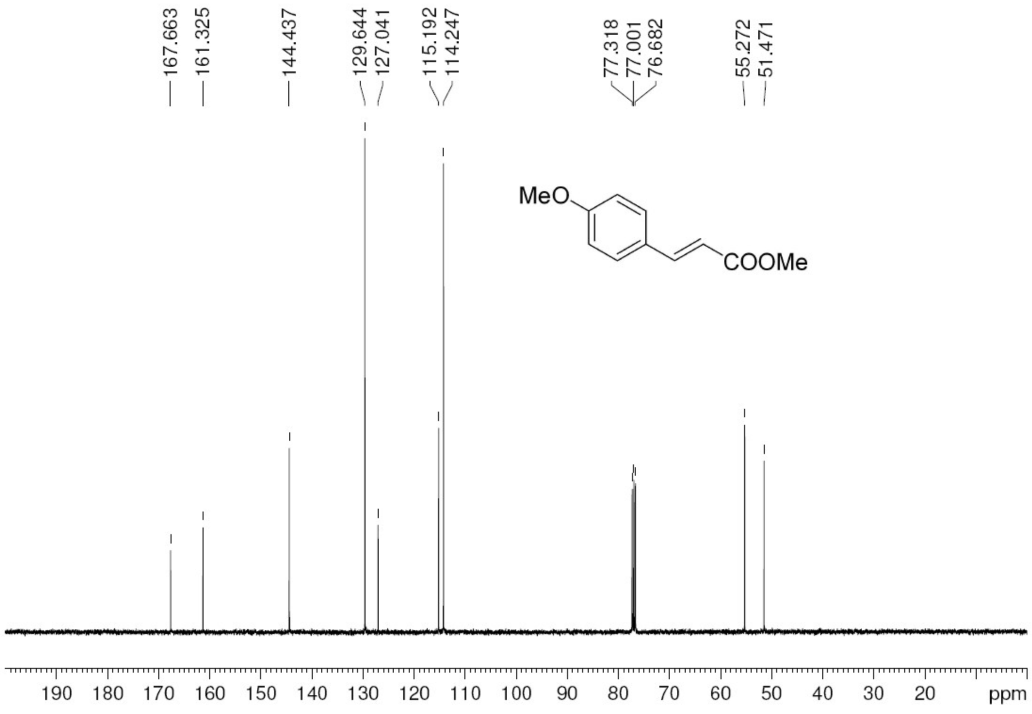
167.663
161.325
144.437
129.644
127.041
115.192
114.247
77.318
77.001
76.682
55.272
51.471
MeO
COOMe
190 180 170 160 150 140 130 120 110 100 90 80 70 60 50 40 30 20
ppm

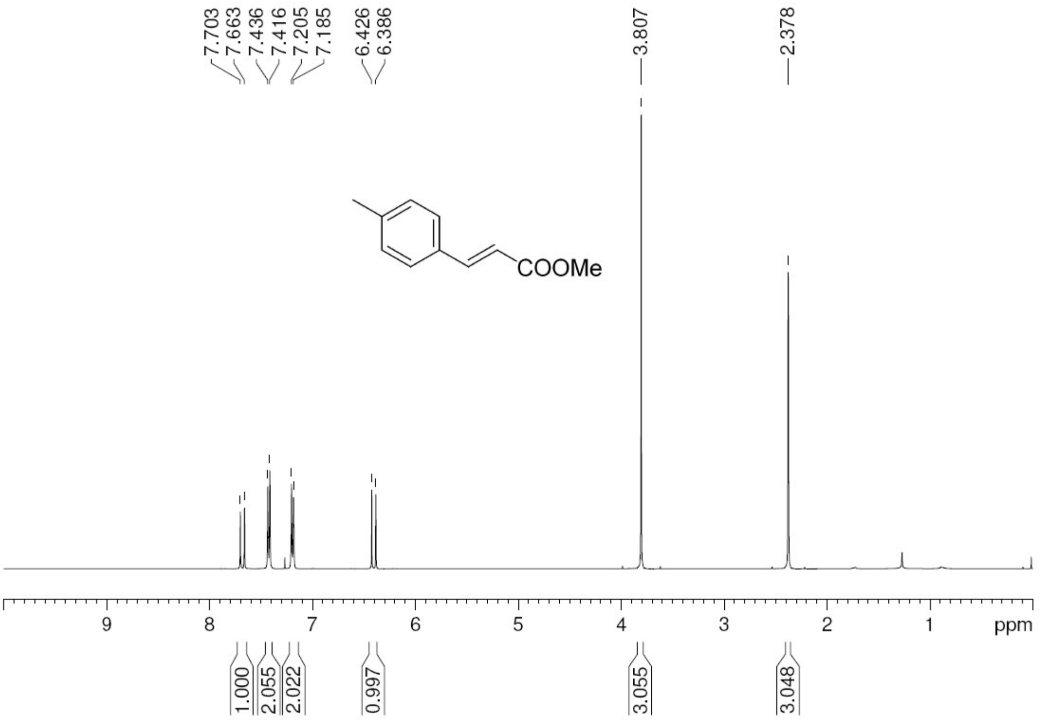

7.703
7.663
7.436
7.416
7.205
7.185
6.426
6.386
3.807
2.378
COOMe
9 8 7 6 5 4 3 2 1 ppm
1.000
2.055
2.022
0.997
3.055
3.048

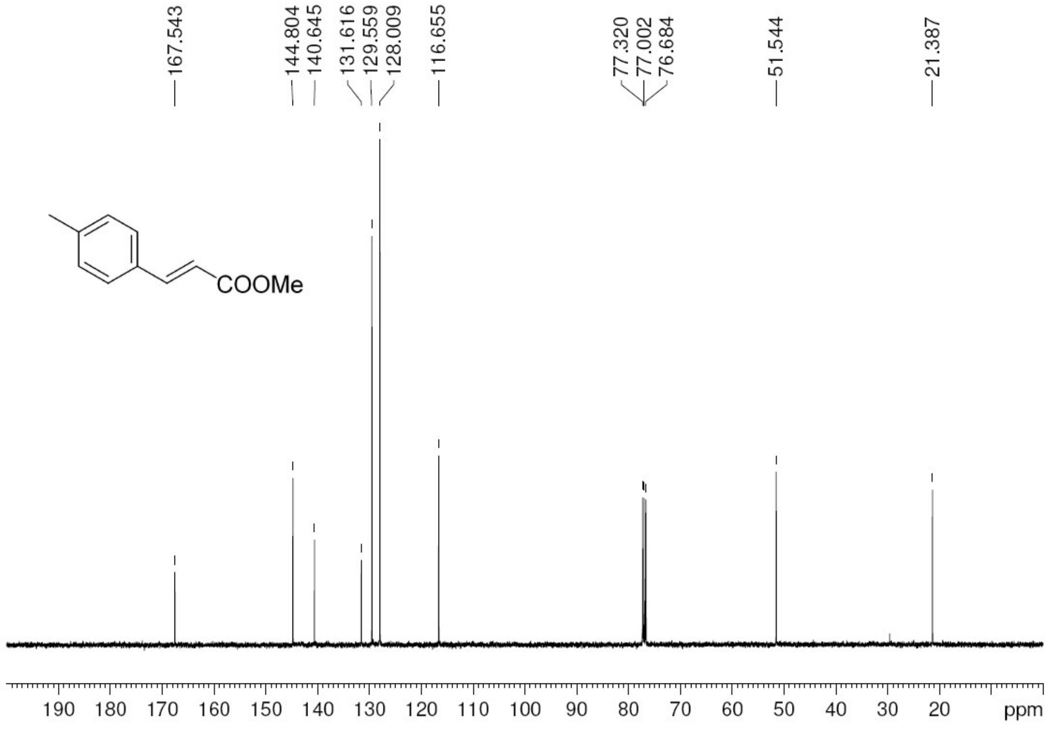

167.543
144.804
140.645
131.616
129.559
128.009
116.655
77.320
77.002
76.684
51.544
21.387
COOMe
190 180 170 160 150 140 130 120 110 100 90 80 70 60 50 40 30 20 ppm

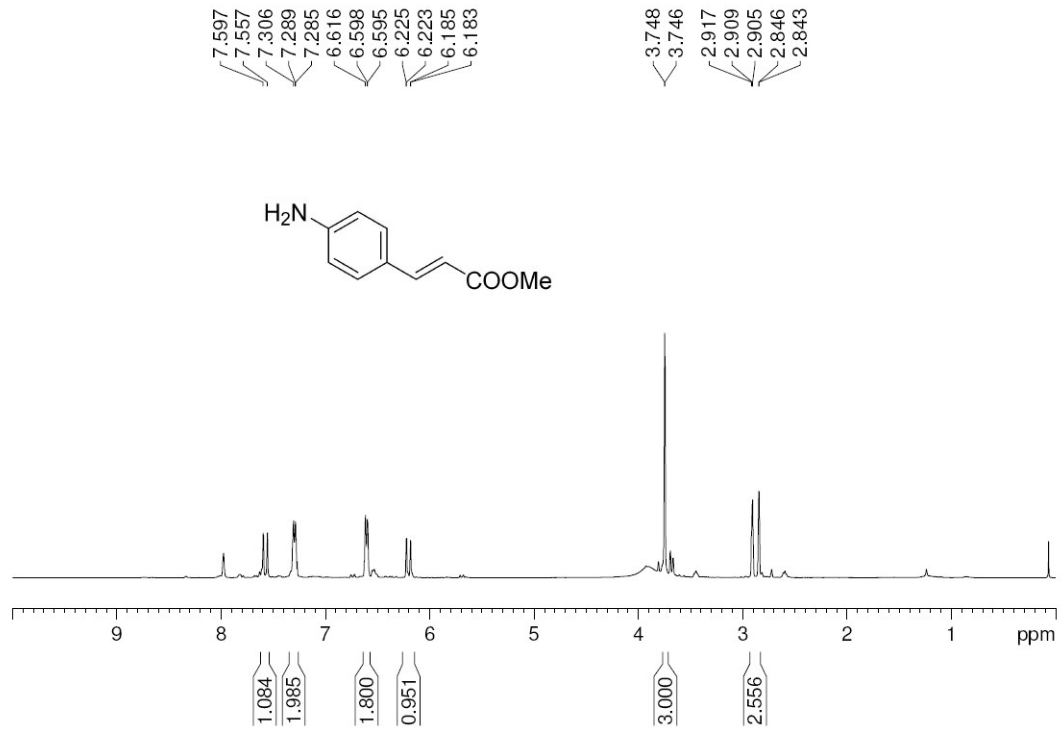

7.597
7.557
7.306
7.289
7.285
6.616
6.598
6.595
6.225
6.223
6.185
6.183
3.748
3.746
2.917
2.909
2.905
2.846
2.843
H2N
COOMe
9
8
7
6
5
4
3
2
1
ppm
1.084
1.985
1.800
0.951
3.000
2.556

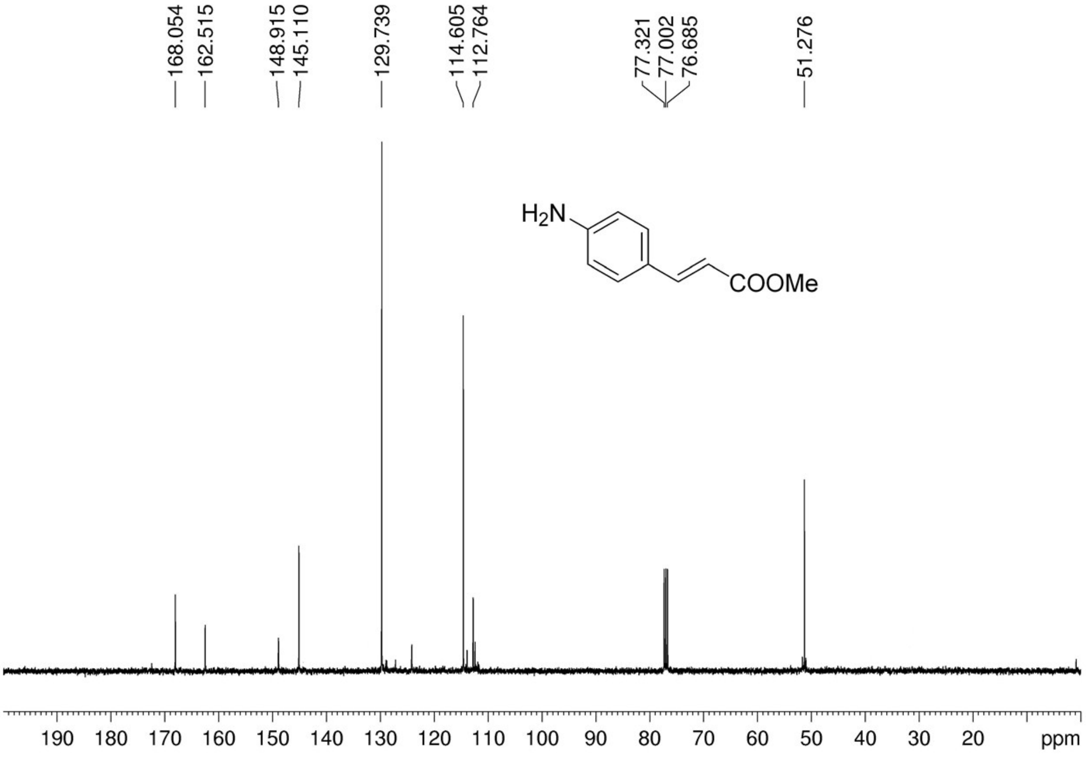

168.054
162.515
148.915
145.110
129.739
114.605
112.764
77.321
77.002
76.685
51.276
H2N
COOMe
190
180
170
160
150
140
130
120
110
100
90
80
70
60
50
40
30
20
ppm

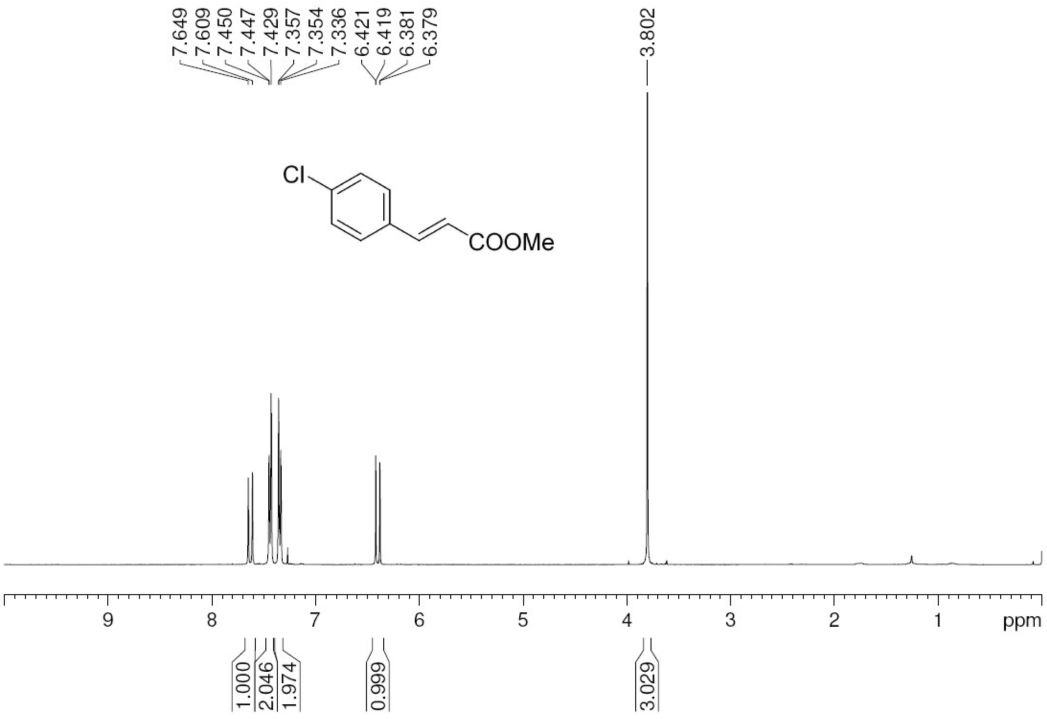
7.649
7.609
7.450
7.447
7.429
7.357
7.354
7.336
6.421
6.419
6.381
6.379
3.802
Cl
COOMe
9
8
7
6
5
4
3
2
1
ppm
1.000
2.046
1.974
0.999
3.029

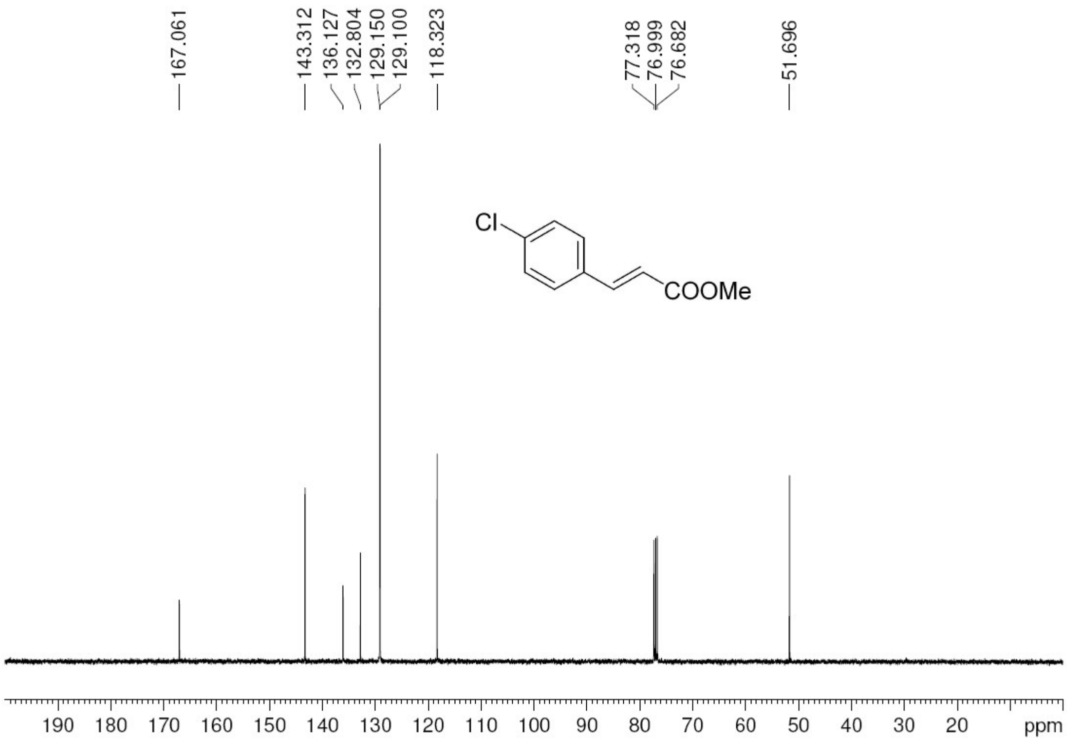
167.061
143.312
136.127
132.804
129.150
129.100
118.323
77.318
76.999
76.682
51.696
Cl
COOMe
190
180
170
160
150
140
130
120
110
100
90
80
70
60
50
40
30
20
ppm

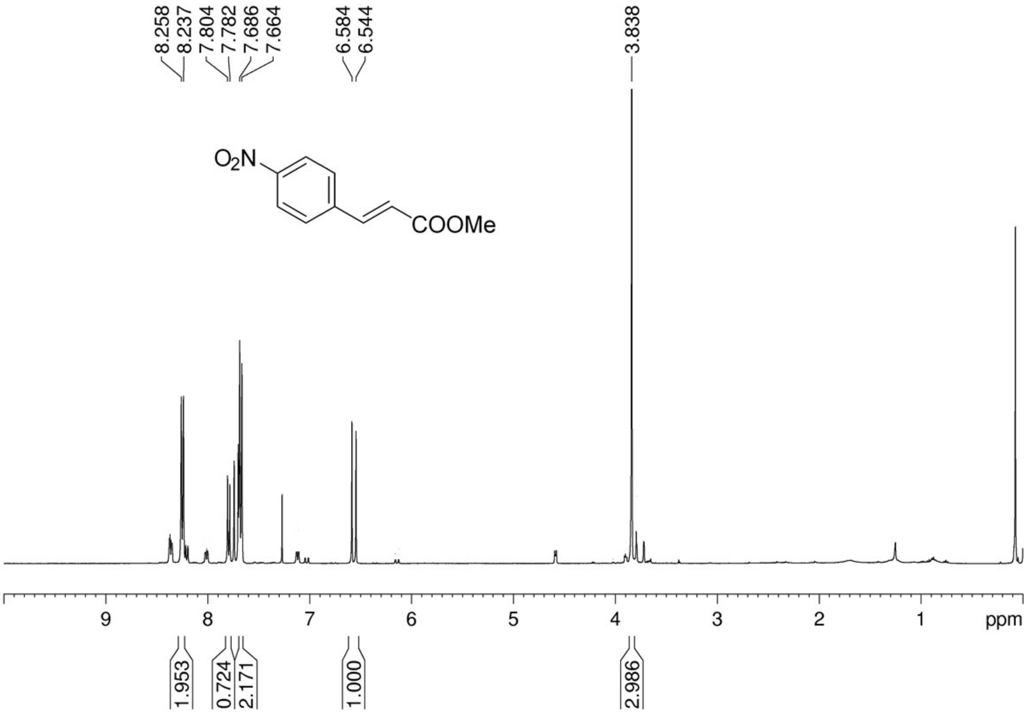

8.258
8.237
7.804
7.782
7.686
7.664
6.584
6.544
3.838
O_2N
COOMe
9 8 7 6 5 4 3 2 1 ppm
1.953
0.724
2.171
1.000
2.986

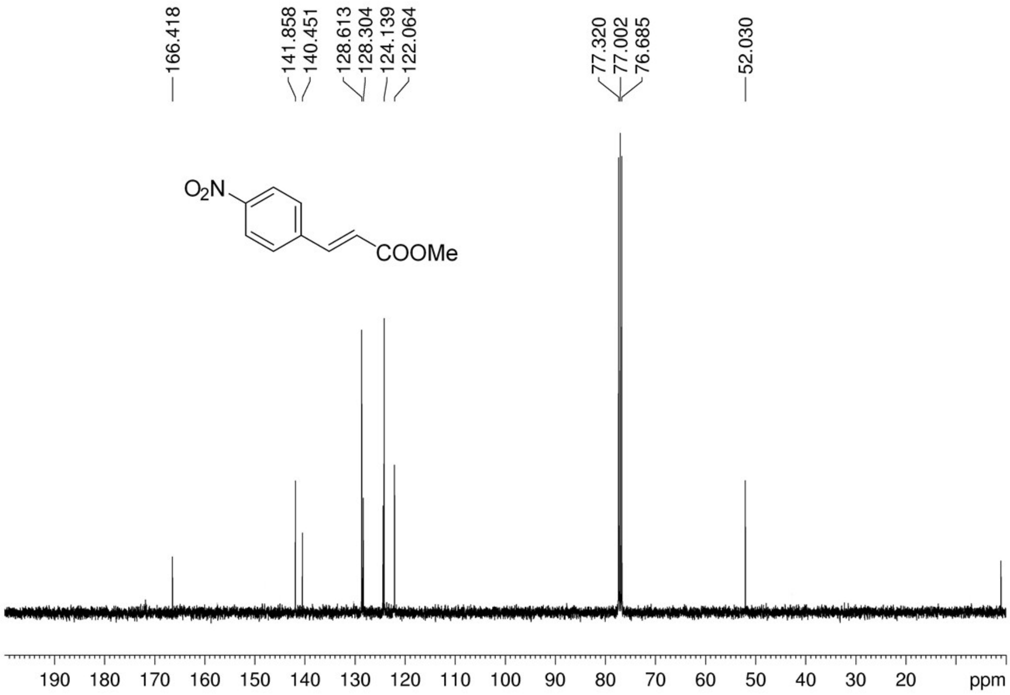

166.418
141.858
140.451
128.613
128.304
124.139
122.064
77.320
77.002
76.685
52.030
O_2N
COOMe
190 180 170 160 150 140 130 120 110 100 90 80 70 60 50 40 30 20 ppm

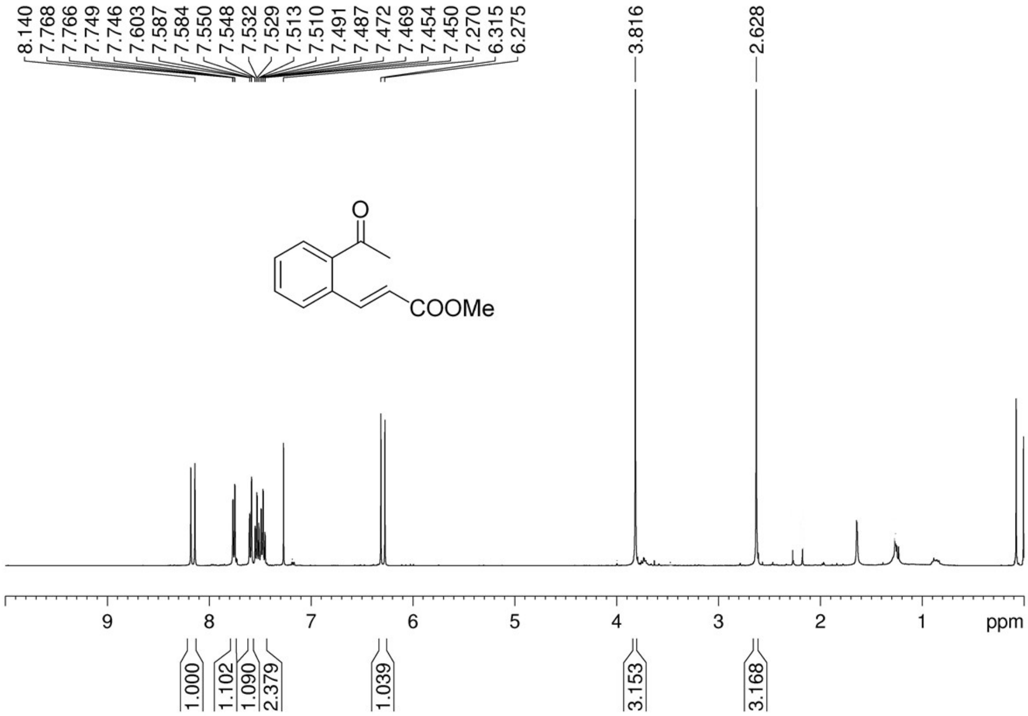
8.140
7.768
7.766
7.749
7.746
7.603
7.587
7.584
7.550
7.548
7.532
7.529
7.513
7.510
7.491
7.487
7.472
7.469
7.454
7.450
7.270
6.315
6.275
3.816
2.628
COOMe
9
8
7
6
5
4
3
2
1
ppm
1.000
1.102
1.090
2.379
1.039
3.153
3.168

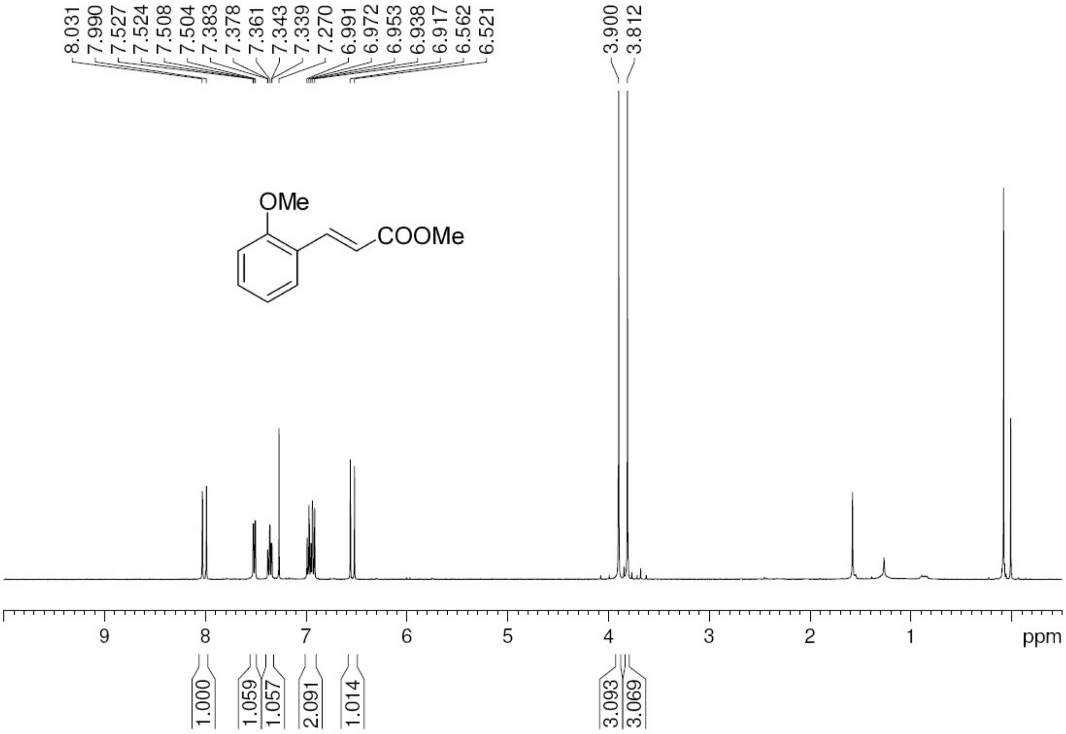
8.031
7.990
7.527
7.524
7.508
7.504
7.383
7.378
7.361
7.343
7.339
7.270
6.991
6.972
6.953
6.938
6.917
6.562
6.521
3.900
3.812
OMe
COOMe
9
8
7
6
5
4
3
2
1
ppm
1.000
1.059
1.057
2.091
1.014
3.093
3.069

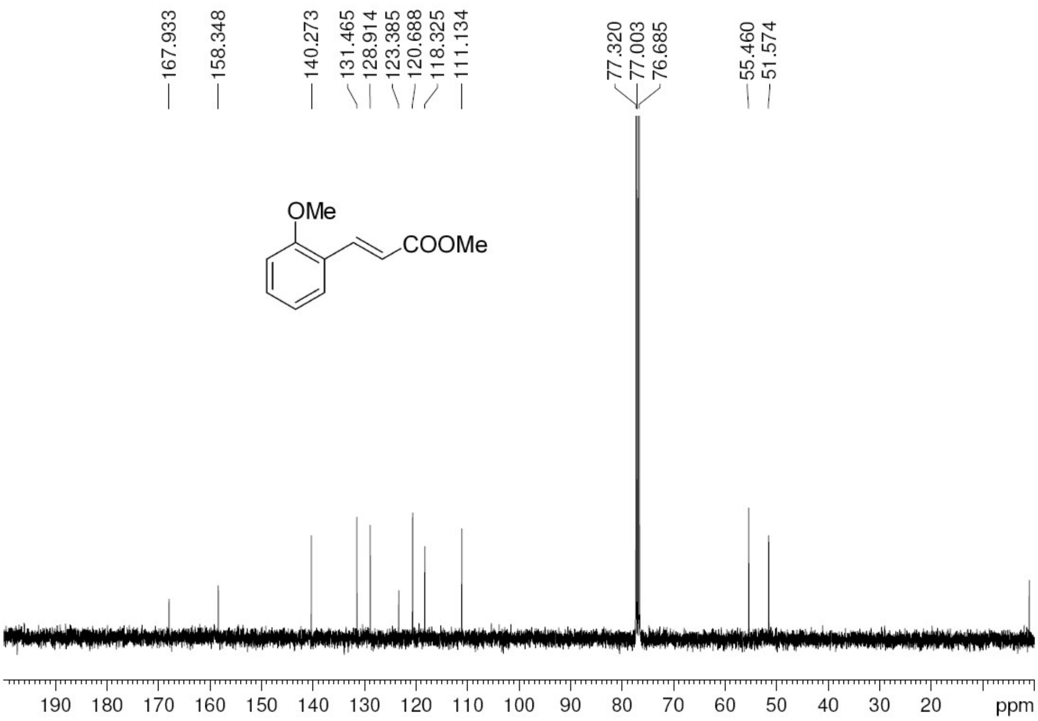
167.933
158.348
140.273
131.465
128.914
123.385
120.688
118.325
111.134
77.320
77.003
76.685
55.460
51.574
OMe
COOMe
190 180 170 160 150 140 130 120 110 100 90 80 70 60 50 40 30 20 ppm

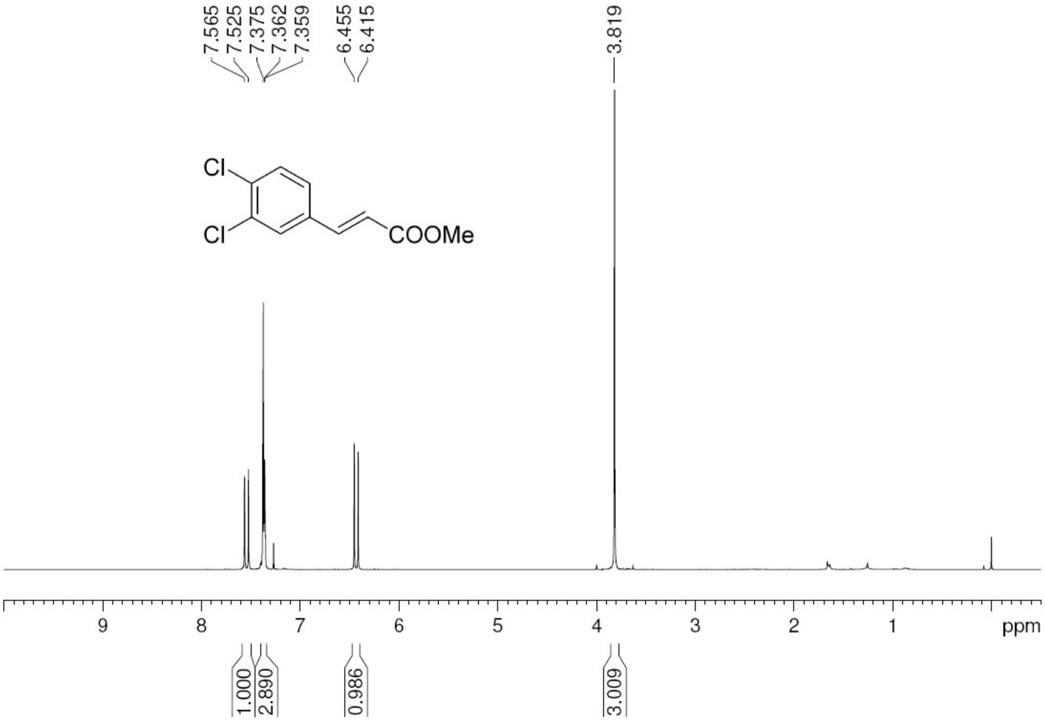
7.565
7.525
7.375
7.362
7.359
6.455
6.415
3.819
Cl
Cl
COOMe
9 8 7 6 5 4 3 2 1 ppm
1.000
2.890
0.986
3.009

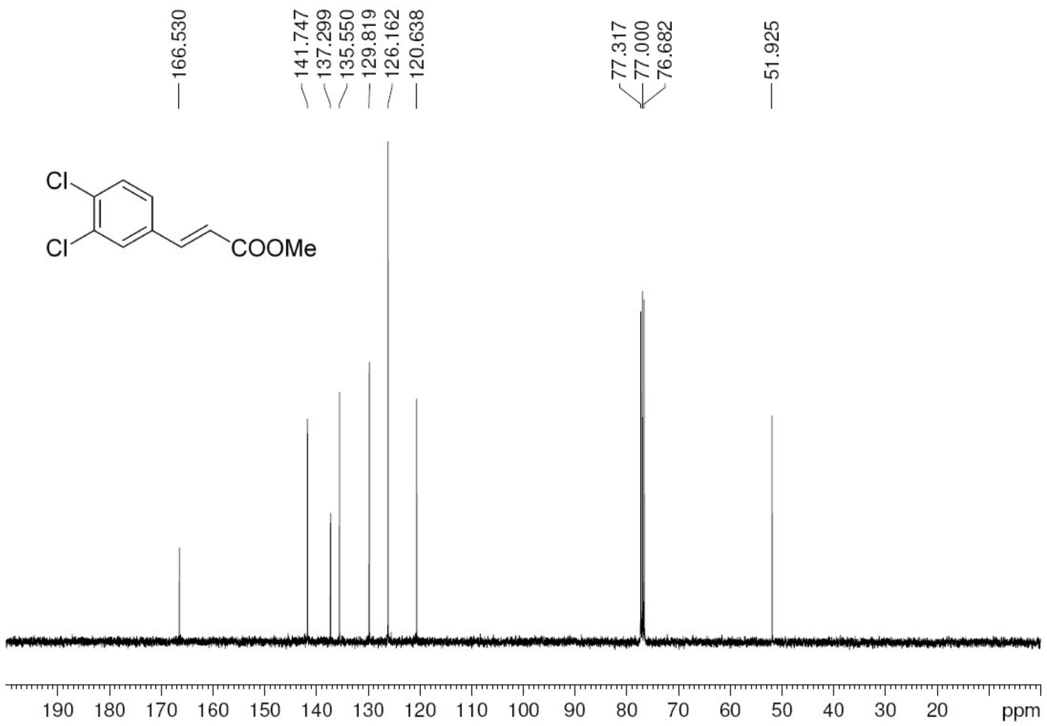
166.530
141.747
137.299
135.550
129.819
126.162
120.638
77.317
77.000
76.682
51.925
Cl
Cl
COOMe
190 180 170 160 150 140 130 120 110 100 90 80 70 60 50 40 30 20 ppm

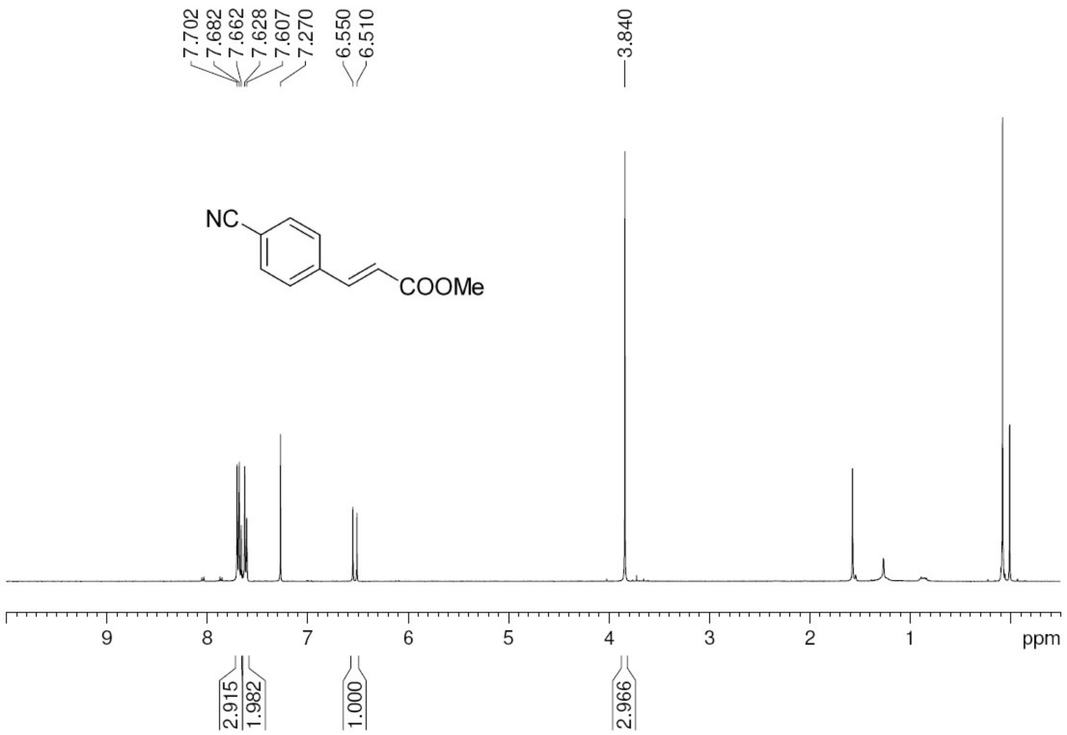
7.702
7.682
7.662
7.628
7.607
7.270
6.550
6.510
3.840
NC
COOMe
9 8 7 6 5 4 3 2 1 ppm
2.915
1.982
1.000
2.966

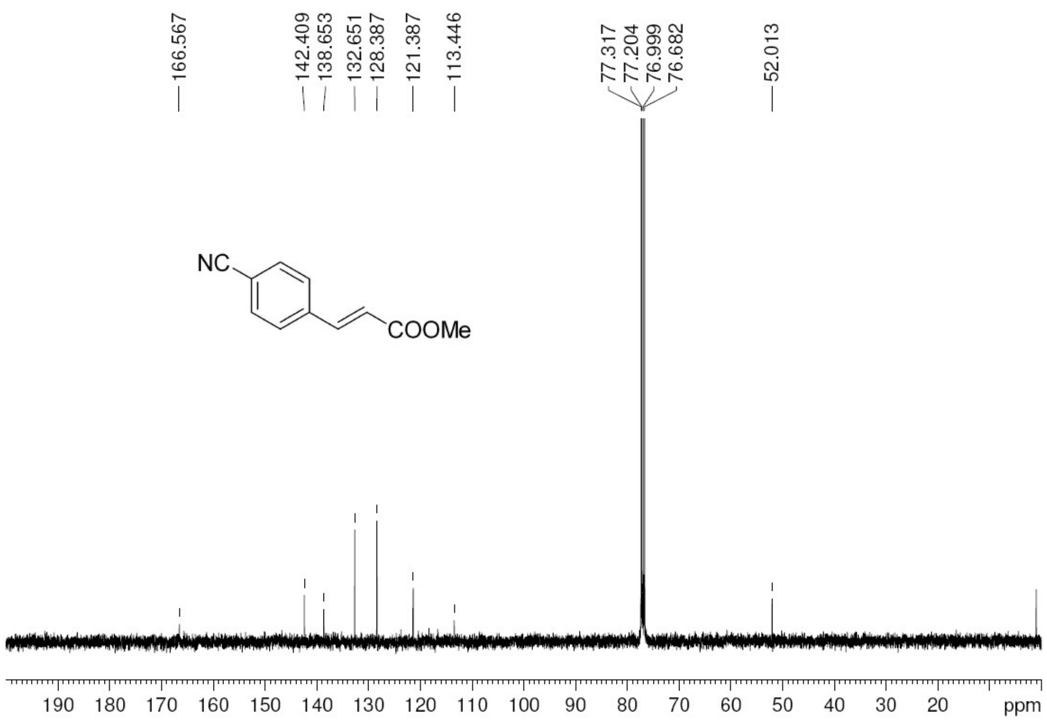
166.567
142.409
138.653
132.651
128.387
121.387
113.446
77.317
77.204
76.999
76.682
52.013
NC
COOMe
190 180 170 160 150 140 130 120 110 100 90 80 70 60 50 40 30 20 ppm

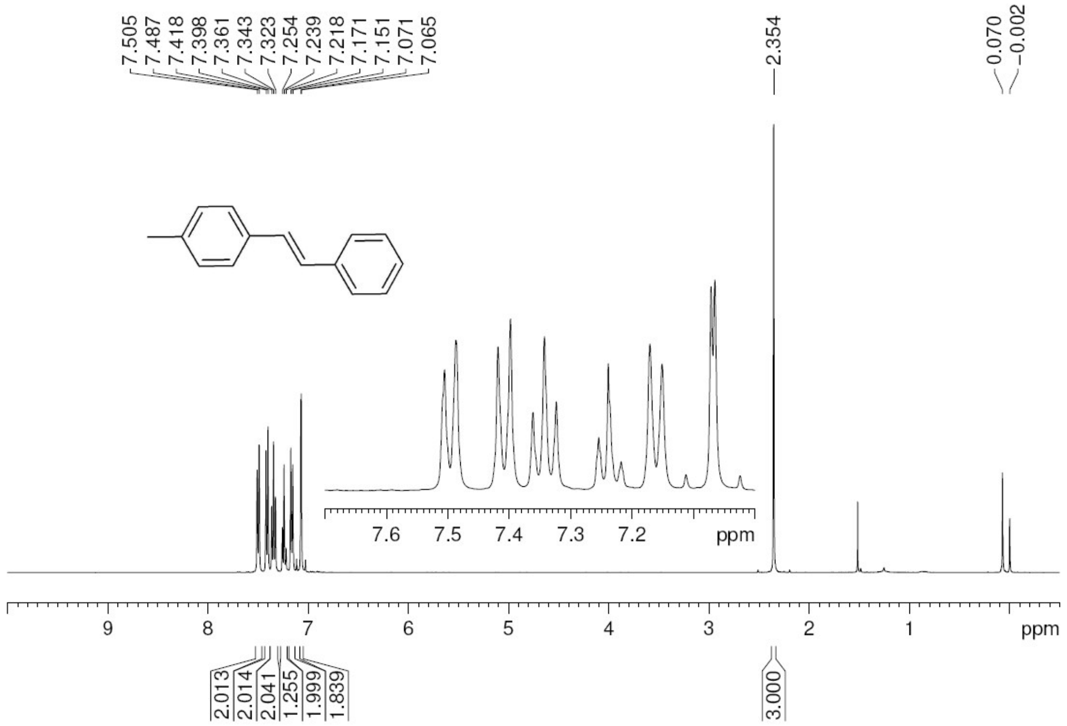
7.505
7.487
7.418
7.398
7.361
7.343
7.323
7.254
7.239
7.218
7.171
7.151
7.071
7.065
2.354
0.070
-0.002
7.6 7.5 7.4 7.3 7.2 ppm
9 8 7 6 5 4 3 2 1 ppm
2.013
2.014
2.041
1.255
1.999
1.839
3.000

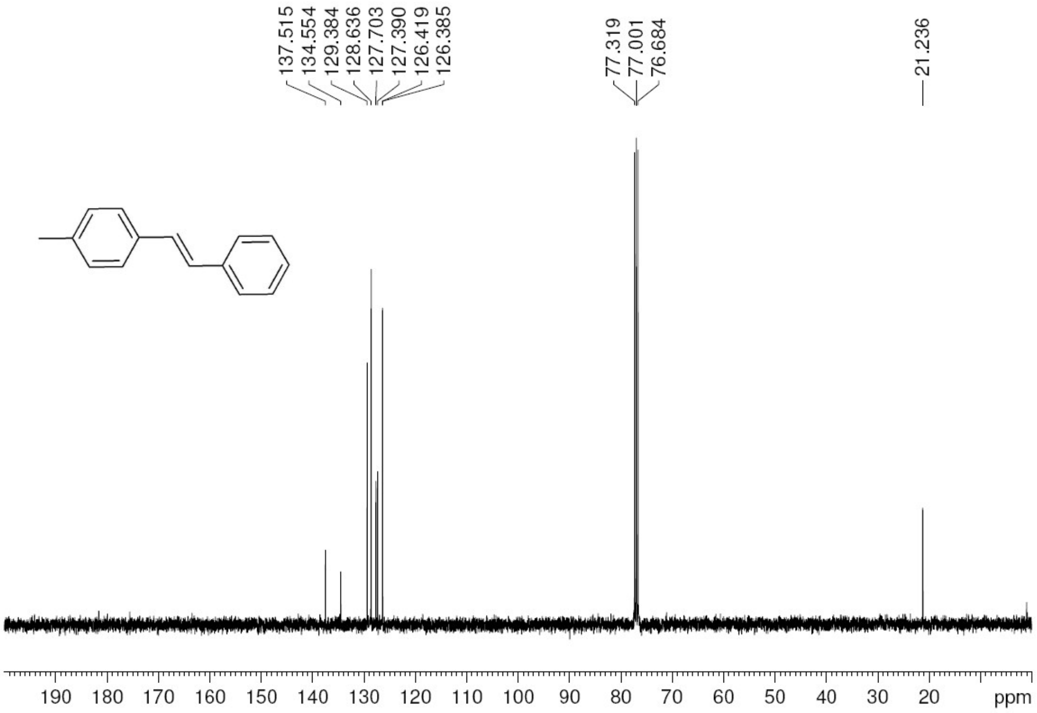

137.515
134.554
129.384
128.636
127.703
127.390
126.419
126.385
77.319
77.001
76.684
21.236
190 180 170 160 150 140 130 120 110 100 90 80 70 60 50 40 30 20
ppm
